# Disperse azo dyes, arylamines and halogenated dinitrobenzene compounds in synthetic garments on the Swedish market

**DOI:** 10.1111/cod.14163

**Published:** 2022-06-10

**Authors:** Josefine Carlsson, Tim Åström, Conny Östman, Ulrika Nilsson

**Affiliations:** ^1^ Department of Materials and Environmental Chemistry Stockholm University Stockholm Sweden

**Keywords:** arylamines, clothing, contact allergy, disperse azo dyes, halogenated dinitrobenzenes, screening, textile dye mix

## Abstract

**Background:**

Azobenzene disperse dyes (azo DDs) are well‐known as textile allergens, but the knowledge of their occurrence in garments is low. The numerous azo DDs and dye components found in textiles constitute a potential health risk, but only seven azo DDs are included in the European baseline patch test series (EBS).

**Objectives:**

To investigate non‐regulated azo DDs and dye components in synthetic garments on the Swedish market.

**Methods:**

High‐performance liquid chromatography/mass spectrometry, gas chromatography/mass spectrometry and computerized data mining.

**Results:**

Sixty‐two azo DDs were detected, with Disperse Red 167:1 occurring in 67%, and 14 other DDs each found in >20% of the garments. Notably, the EBS dyes were less common, three even not detected, while arylamines were frequently detected and exceeded 1 mg/g in several garments. Also, halogenated dinitrobenzenes were identified in 25% of the textiles.

**Conclusion:**

Azo DDs and dye components, in complex compositions and with large variations, occurred frequently in the synthetic garments. The arylamines were shown to occur at much higher levels compared to the azo DDs, suggesting the former constitute a potentially higher health risk. The role of arylamines and halogenated dinitrobenzenes in textile allergy has to be further investigated.

## Graphical abstract

- Disperse Red 167:1 was the most frequent among 62 detected azo dyes in the investigated synthetic garments. The azo dyes in the European baseline patch test series were less common.
- Arylamines were detected at much higher levels than azo dyes.
- Skin‐sensitizing 1‐chloro‐2,4‐dinitrobenzene (2,4‐DNCB) was identified in 6% of the garments.

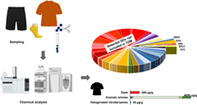

## 1 INTRODUCTION

**FIGURE 1:**
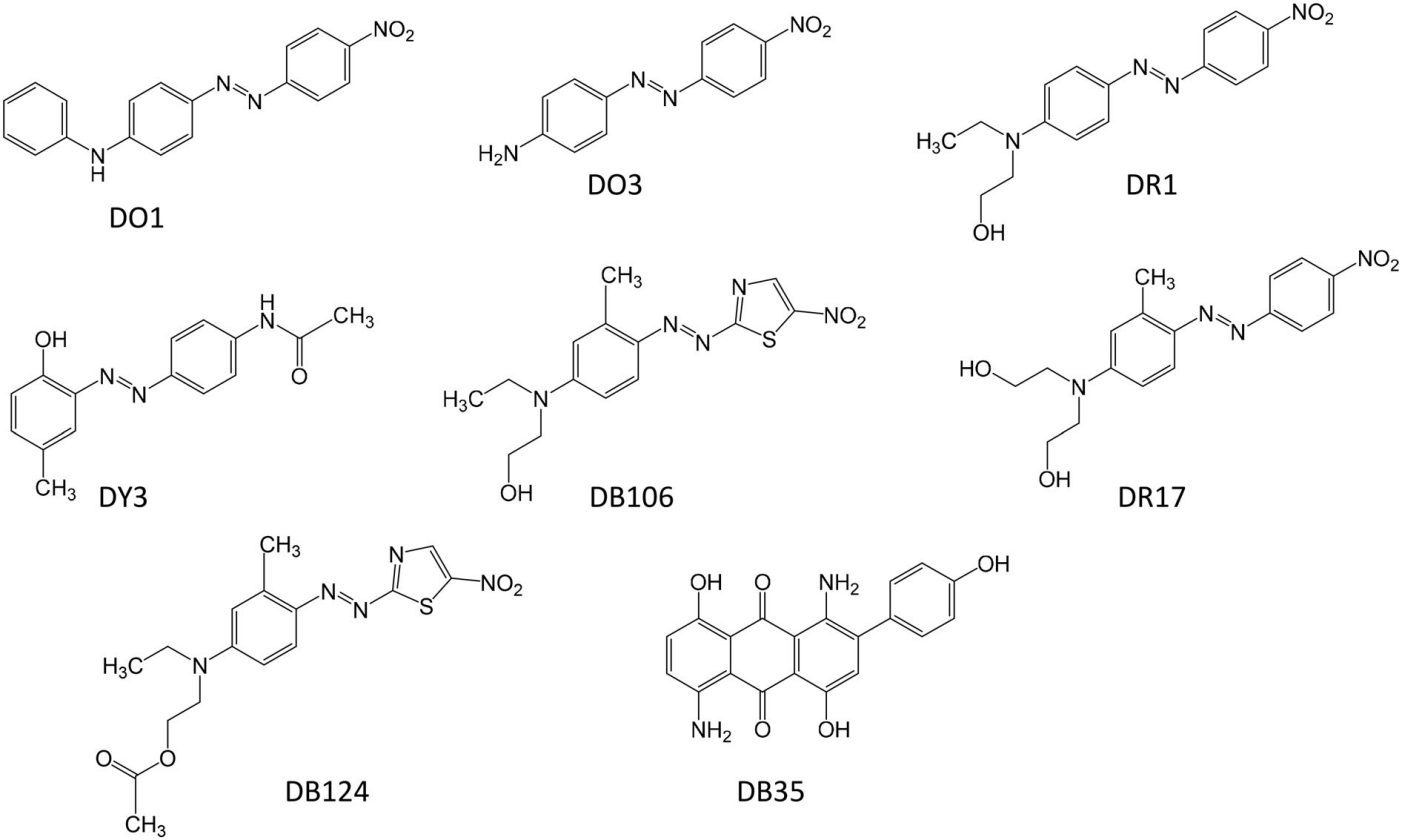
Disperse dyes (DDs) in the textile dye mix (TDM). DO1, Disperse Orange 1; DO3, Disperse Orange 3; DR1, Disperse Red 1; DR17, Disperse Red 17; DY3, Disperse Yellow 3; DB106, Disperse Blue 106; DB124, Disperse Blue 124; DB35, Disperse Blue 35. DB35 is of anthraquinone type, all other DDs shown are azo DDs

Contact allergy towards textiles is commonly caused by chemical compounds in the fabric, such as dyes or formaldehyde resins, while more seldom by the fibre itself.^1^, ^2^, ^3^ Disperse dyes (DDs) are considered to be the most important textile allergens and DDs of both azo‐ and anthraquinone types are well‐known strong skin sensitizers.^3^ DDs are used to colour synthetic fibres such as polyester, polyamide, acetate and in some cases nylon or blends of those. Due to the lipophilic characteristics of DDs, they diffuse into the hydrophobic fibres and typically heat and dispersive agents are applied to speed up the process.^4^ Consequently, the DD molecules are not covalently bound to the textile fibres and in skin‐close garments, this may lead to migration to the skin, facilitated by sweat, heat and friction. The dye molecules can be either absorbed by the skin and then metabolized to protein‐reactive skin‐sensitizing substances, or activated by skin bacteria to allergenic arylamines before skin absorption.^5^, ^6^, ^7^ Those azobenzene disperse dyes (azo DDs) that may be reduced to any of the 22 known mutagenic and carcinogenic arylamines in concentrations above 30 μg/g are prohibited for use in textiles within the European Union according to REACH regulation Annex XVII.^8^ There is also voluntary eco‐labelling, such as the OEKO‐TEX®, which further restricts the use of some allergenic dyes.^9^ However, there are few regulations today considering a large number of azo DDs used in the textile industry. Very little knowledge, as well as information, is available regarding the content of these compounds in garments. Data on the reported prevalence of contact allergy to DDs between 1990 and 2012 have been evaluated in a review from 2012.^10^ From these data, 26 DDs were identified as allergens. However, in the review, it was pointed out that these compounds are only a tiny fraction of all possible allergenic DDs. Also, the relevant contact allergens among DDs in textile allergy can be very difficult to identify since the dyestuff used for textile colouring are often highly impure.^11^, ^12^ It has been shown previously that the DDs used for patch testing may contain numerous pollutants.^13^, ^14^, ^15^ A textile dye mix (TDM) with a totally of 6.6% (wt/wt) of eight DDs, the azo compounds Disperse Orange 1 and 3, Disperse Red 1 and 17, Disperse Yellow 3, Disperse Blue 106 and 124 and the anthraquinone dye Disperse Blue 35 (Figure 1), is now included in the European baseline series (EBS) for patch testing.^16^, ^17^, ^18^, ^19^, ^20^, ^21^ However, a study already in 2012 indicated that these TDM dyes are not frequent in textile garments.^11^ The majority of azo DDs used within the global textile industry are non‐regulated, although they may contain or release arylamines of toxicological concern, with health effects such as skin sensitization,^22^ genotoxicity,^23^ carcinogenicity^22^, ^23^ and mutagenicity.^24^ It has also been shown that arylamines with suspected toxic effects can be formed after reductive treatment of the dye content in clothes.^22^, ^25^, ^26^ Such arylamines have been detected at levels exceeding 0.2 mg/g in underwear garments from the US market.^27^ Furthermore, an investigation of textiles from the Swedish market, detected arylamines at levels up to 0.5 mg/g.^28^

The work aimed to investigate synthetic garments from the Swedish retail market and identify the most frequently occurring non‐regulated azo DDs and dye components. Screening of a large number of suspect DDs based on published information and available reference standards was performed with high‐performance liquid chromatography/high‐resolution mass spectrometry (HPLC/HRMS) and quantification with HPLC/triple‐stage quadrupole (TSQ) MS. For semivolatile components, gas chromatography/mass spectrometry (GC/MS) was used.

## 2 METHODS

### 2.1 Chemicals

Methanol, acetonitrile (both HPLC grade), dichloromethane (puriss.) and ammonium acetate (analytical reagent) were purchased from Riedel‐de Haën. Ultrapure water with a resistivity of 18.2 MΩ cm was obtained with a Synergy 185 system from Millipore. The purities and suppliers of the standard compounds are shown in Supporting Information (Table S1).

### 2.2 Samples and sample preparation

Eighty‐two low‐ to mid‐price common textile garments were purchased from different suppliers. For 11 of the garments, the manufacturing country was unknown, while the rest were imported from different countries. The colours, materials and manufacturing countries are listed in Table S2. The selected textiles were all skin‐close garments, such as T‐shirts, socks and so forth, made of more than 70% polyester fibres. All analysed textiles were one‐coloured, except one. The latter had two parts of different colours that were analysed separately. Thus, 83 samples were altogether subject to analysis. About 1 g of textile was cut into pieces of approx. 4 × 4 mm and put into a 15‐ml glass tube, and extracted with 2 × 6 ml of dichloromethane using ultrasonication (10 min, 100% amplitude at 50°C, Sonorex Digital 10P; Bandeline Electronic). This part of the sample preparation was the same for all analyses, while the following procedures differed slightly between the applied analytical methods, as described below.

Before analysis with HPLC/HRMS, a volume of 200 μl of water was added to the pooled dichloromethane extract and the resulting volume was reduced to 200 μl under a gentle flow of nitrogen at 35°C. A volume of 800 μl of methanol was then added, followed by syringe filtration into a vial. A volume of 5 μl of this solution was injected into the HPLC/HRMS.

The sample preparation for HPLC/TSQ MS analysis was similar to for HPLC/HRMS, except that quinoline‐d_7_, 3‐nitroaniline‐d_4_, 4‐nitroaniline‐^15^N_2_ and Sudan‐I‐d_5_ were added as internal standards (IS) before the ultrasonic extraction. A volume of 10 μl of the final sample solution was injected into the HPLC/TSQ MS. The recoveries from the sample preparation for 22 DDs measured by HPLC/TSQ MS are given in Table S3.

For GC/MS analysis and quantification of arylamines and halogenated dinitrobenzene compounds, quinoline‐d_7_, 3‐nitroaniline‐d_4_, 4‐nitroaniline‐^15^N_2_ and Sudan‐I‐d_5_ were added as IS before the ultrasonic extraction. After filtration, the dichloromethane solvent was then evaporated to 1 ml, of which 1 μl was injected into the GC/MS. The recoveries from the sample preparation for arylamines and halogenated dinitrobenzenes are given in Table S4.

### 2.3 Instrumental analysis

A full description of the entire analytical methodology can be found in Supporting Information. To summarize, HPLC/HRMS was used for screening of DDs, including detection and identification, but not quantification. For quantification, HPLC/TSQ MS and reference compounds were used. For the semivolatile classes of compounds, that is, arylamines and halogenated dinitrobenzenes, GC/MS and reference compounds were used for both identification and quantification.

#### 2.3.1 HPLC/HRMS

**FIGURE 2:**
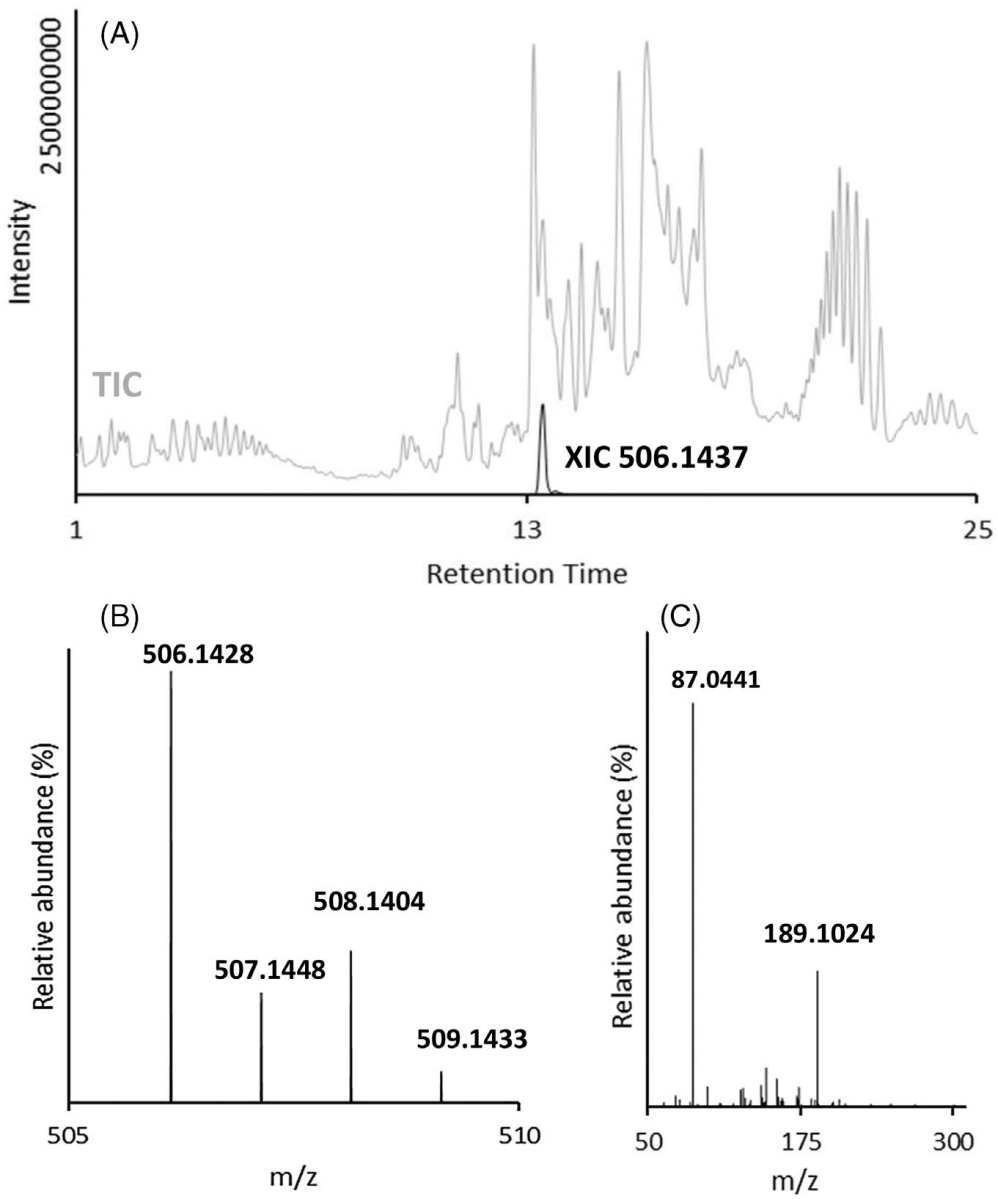
HPLC/HRMS analysis—Example of first stage identification of Disperse Red 167:1 in a sample extract from a black T‐shirt made of 100% recycled polyester fibres (sample S‐52). (A) shows the total ion chromatogram (TIC) as a grey line. At retention time (RT) 13.4 min the extracted ion chromatogram (XIC, exact mass *m*/*z* 506.1437, black line) shows the peak identified as Disperse Red 167:1. The full MS isotopic pattern of this peak (B) is matching with the theoretical spectrum of Disperse Red 167:1, the MS/MS fragmentation pattern is shown in (C). Disperse Red 167:1 is identified by HPLC retention time, full MS accurate mass isotopic pattern, as well as MS/MS accurate mass fragmentation pattern

We have recently published a suspect screening workflow with HPLC/HRMS for identification purposes including data mining.^29^ In the present work, this was used for screening of more than 120 different DDs and some related dye components, suspected to be present in synthetic garments based on both published information regarding usage in industry^22^ and several available reference standards (Table S5). The instrument used for this suspect screening was a UHPLC/electrospray (ESI)‐Orbitrap HRMS instrument (Q Exactive HF; Thermo Fisher Scientific). A first stage identification of DDs was performed, utilizing accurate mass, MS/MS spectra, isotopic pattern, and experimental HPLC retention times from those predicted, by a model shown in Supporting Information S6. No quantification was performed at this stage. Figure 2 illustrates the HPLC/HRMS identification workflow.

#### 2.3.2 HPLC/TSQ MS

HPLC/TSQ MS was used for quantification of the 14 DDs for which reference compounds were available. In this case, a Waters Acquity I‐Class UPLC system (Waters), coupled to a Xevo TQ‐S triple quadrupole MS instrument equipped with an ESI source was used. The instrumental settings are given in Table S7. No matrix effects on the ionization could be observed and the instrumental response was linear (*R* ^2^ > 0.99) within the measured range of 0.02 to 1.6 ng amount injected. The relative standard deviation was within the range of 1%–13% (*n* = 3) and the method limit of quantification (S/N = 10) was determined to be 0.7–20.5 ng/g of textile, depending on the dye.

#### 2.3.3 GC/MS

**FIGURE 3:**
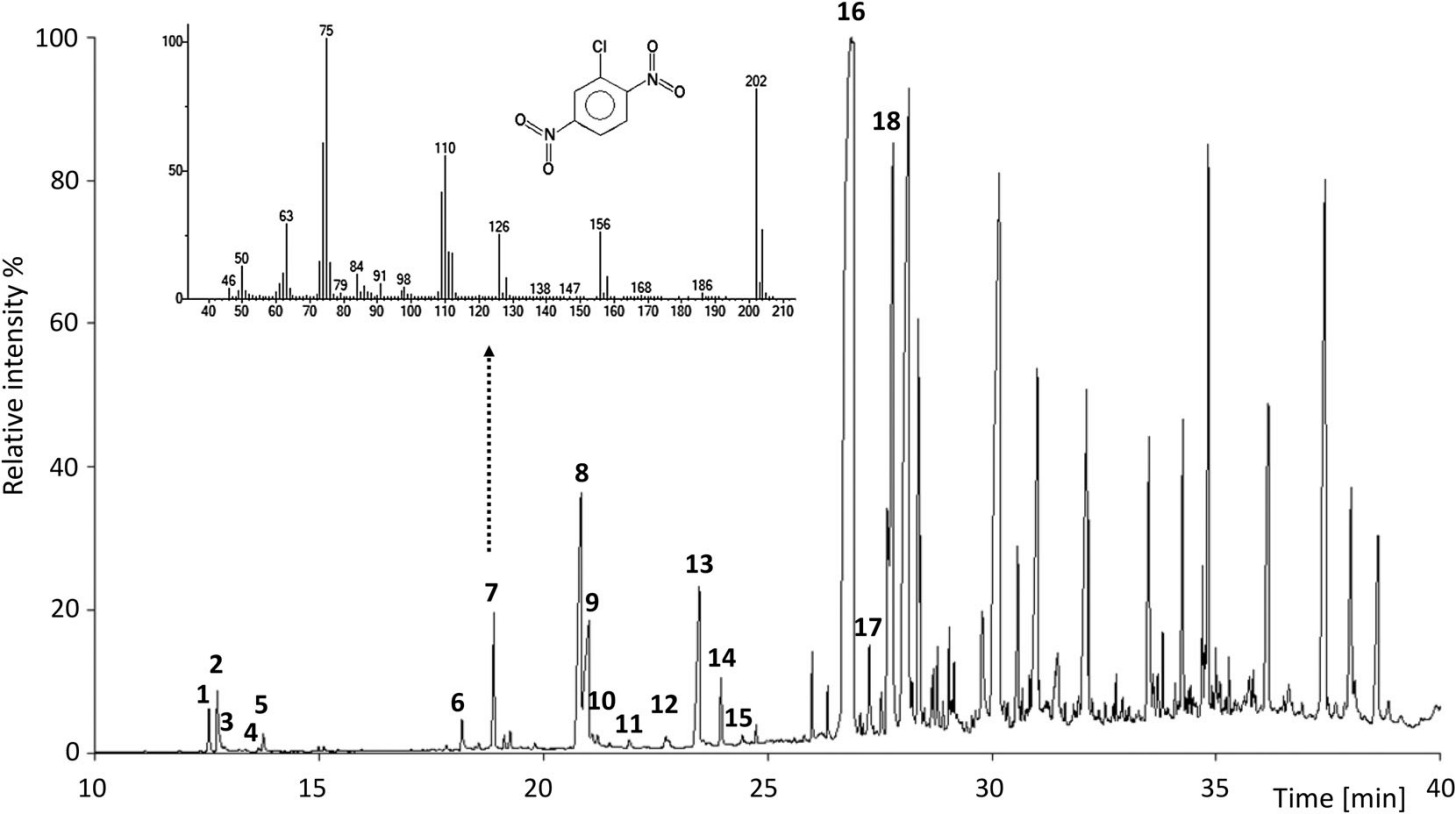
A GC/MS chromatogram, from retention time 10–40 min, of an extract from the same black t‐shirt made of 100% recycled polyester fibres (sample S‐52) as in Figure 2. The numbered peaks in the chromatogram corresponds to: (1) 1‐chloro‐4‐nitrobenzene, (2) quinoline‐d_7_ (IS), (3) quinoline, (4) 4‐chloro‐2‐nitrophenol, (5) indanone (IS), (6) 3‐nitroaniline‐d_4_ (IS), (7) 1‐chloro‐2,5‐dinitrobenzene, (8) 4‐chloro‐2‐nitroaniline, (9) 4‐nitroaniline, (10) 1‐bromo‐3,5‐dinitrobenzene, (11) dichloro‐dinitrobenzene*, (12) chloro‐nitroaniline*, (13) 2‐chloro‐4‐nitroaniline, (14) 2,6‐dichloro‐4‐nitroaniline, (15) bromo‐chloro‐dinitrobenzene*, (16) 6‐chloro‐2,4‐dinitroaniline, (17) 2,4‐dinitroaniline, (18) 2‐bromo‐4,6‐dinitroaniline. For the compounds marked with *, the exact positions of the substituents are not known since standard compounds were not available. IS, internal standard. The inset shows the identification of 1‐chloro‐2,5‐dinitrobenzene by its mass spectrum and GC retention time compared to the reference compound

For GC/MS of arylamines and halogenated dinitrobenzenes, an Agilent 5975C MSD was used, equipped with a 6890N GC, a 7693 autosampler, and a PTV‐injector (Agilent Technologies). The analysis was performed in full scan mode with electron ionization (EI) at 70 eV. The instrumental settings and calibration data are given in Table S8. The method quantification limits (S/N = 10) were in the range of 9.9–319.2 ng/g of textile, depending on the compound. Figure 3 shows a GC/MS chromatogram representing an extract from a black T‐shirt made of 100% polyester.

## 3 RESULTS

### 3.1 Azo DDs in clothing garments

**FIGURE 4:**
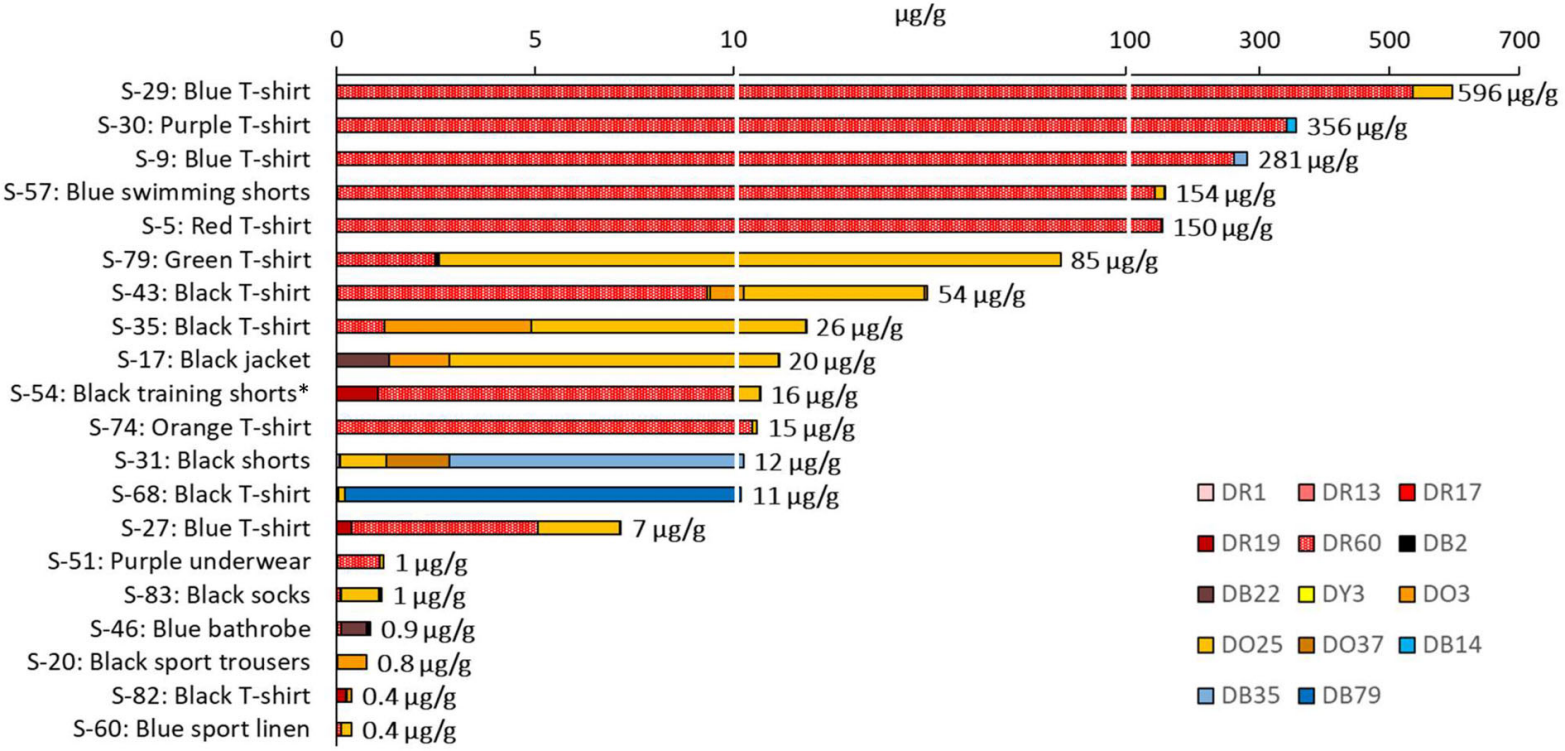
Quantified DDs in selected samples (*n* = 20). Abbreviations within brackets. Disperse Red 1 (DR1), Disperse Red 13 (DR13), Disperse Red 17 (DR17), Disperse Red 19 (DR19), Disperse Black 2 (DB2), Disperse Brown 22 (DB22), Disperse Yellow 3 (DY3), Disperse Orange 3 (DO3), Disperse Orange 25 (DO25), Disperse Orange 37 (DO37), Disperse Blue 14 (DB14), Disperse Blue 35 (DB35), Disperse Blue 79 (DB79). * made of 100% recycled polyester. A complete list of the investigated garments with sample numbers (S‐X) and textile materials are given in Table S2

A selection of 20 garment samples with the highest detected levels of DDs are shown in Figure 4. A complete list of all the investigated textiles can be found in Table S2. Although the aim was to focus on the azo type of DDs, we included several anthraquinone DDs: Disperse Blue 35 since it is included in TDM, and Disperse Red 60 and Disperse Blue 14 since reference compounds were available. In total, 65 dyes (62 azo dyes) were identified in this screening, of which 11 azo dyes and 3 anthraquinone dyes were confirmed with reference compounds. For the majority of the dyes screened for, there were no reference compounds available. Identification was considered sufficient without reference compound by using a combination of accurate mass, MS/MS spectra, isotopic pattern, and HPLC retention time predicted from logP values. The ten most frequently detected dyes in the investigated clothing are listed in Table 1. Only one TDM dye is included among the most frequent dyes, Disperse Orange 3 (Figure 1 and Table 1). The TDM dyes Disperse Red 1 and 17, Disperse Yellow 3 and Disperse Blue 35 were all detected in less than 10%. Neither of the TDM dyes Disperse Orange 1, Disperse Blue 106 or Disperse Blue 124 could be detected in any of the textiles.

**TABLE 1:** The 10 most frequently occurring DDs in 82 synthetic garments

Disperse dye (DD)	Frequency (%)	Type	Possible dye precursor/cleavage product
Disperse Red 167:1	67	Azo	2‐Chloro‐4‐nitroaniline
			((3‐Acetamido‐4‐aminophenyl)azanediyl) bis(ethane‐2,1‐diyl) diacetate
Disperse Red 343	49	Azo	2‐Amino‐5‐methyl isophthalonitrile
			N‐(2‐amino‐5‐(diethylamino)phenyl) methanesulfonamide
Disperse Red 311	48	Azo	2,4‐Dinitroaniline
			Dimethyl 3,3′‐((3‐acetamido‐4‐amino phenyl)azanediyl) dipropionate
Disperse Red 153	46	Azo	5,7‐Dichlorobenzo[d]thiazol‐2‐amine
			3‐((4‐Aminophenyl)(ethyl)amino) propanenitrile
Disperse Red 74	44	Azo	4‐Nitroaniline
			((3‐Acetamido‐4‐aminophenyl)azanediyl) bis(ethane‐2,1‐diyl) diacetate
Disperse Orange 25	39	Azo	4‐Nitroaniline
			3‐((4‐Aminophenyl)(ethyl)amino) propanenitrile
Disperse Red 60	38	Anthraquinone	–
			–
Disperse Blue 165	27	Azo	2‐Amino‐3,5‐dinitrobenzonitrile
			N‐(2‐amino‐5‐(dipropylamino)phenyl) acetamide
Disperse Orange 3	26	Azo	1,4‐Phenylenediamine
			4‐Nitroaniline
Disperse Blue 291 (Cl)	26	Azo	6‐Chloro‐2,4‐dinitroaniline
			N‐(2‐amino‐5‐(diethylamino)‐4‐methoxy phenyl)acetamide

Disperse Orange 37, listed by OEKO‐TEX® as a skin allergen, was detected in only 10% of the garments.^9^ A complete list of all detected dyes is given in Table S9. Concentration ranges of quantified DDs are given in Supporting Information S10.

### 3.2 Arylamines and halogenated dinitrobenzene compounds in clothing garments

**FIGURE 5:**
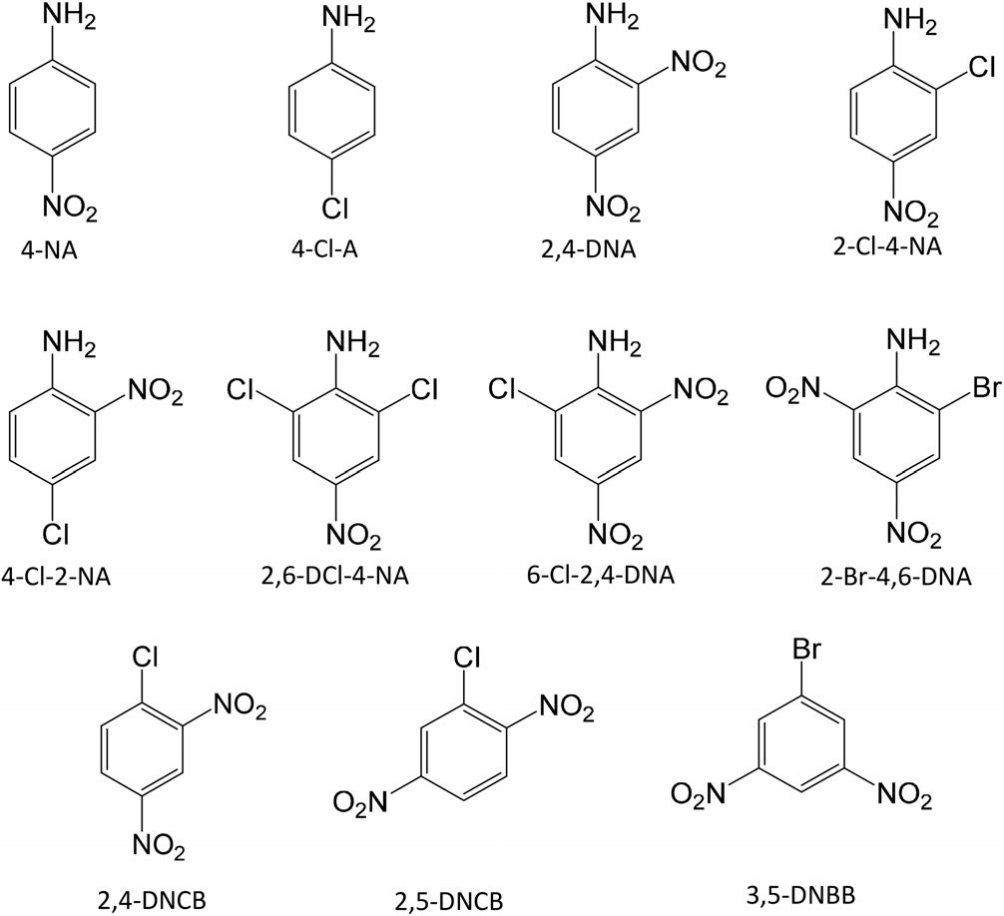
Arylamines detected in more than 10% of the investigated garments, and identified halogenated dinitrobenzenes. 4‐NA, 4‐nitroaniline; 4‐Cl‐A, 4‐chloroaniline; 2,4‐DNA, 2,4‐dinitroaniline; 2‐Cl‐4‐NA, 2‐chloro‐4‐nitroaniline; 4‐Cl‐2‐NA, 4‐chloro‐2‐nitroaniline; 2,6‐DCl‐4‐NA, 2,6‐dichloro‐4‐nitroaniline; 6‐Cl‐2,4‐DNA, 6‐chloro‐2,4‐dinitroaniline; 2‐Br‐4,6‐DNA, 2‐bromo‐4,6‐dinitroaniline; 2,4‐DNCB, 1‐chloro‐2,4‐dinitrobenzene; 2,5‐DNCB, 1‐chloro‐2,5‐dinitrobenzene; 3,5‐DNBB, 1‐bromo‐3,5‐dinitrobenzene

**FIGURE 6:**
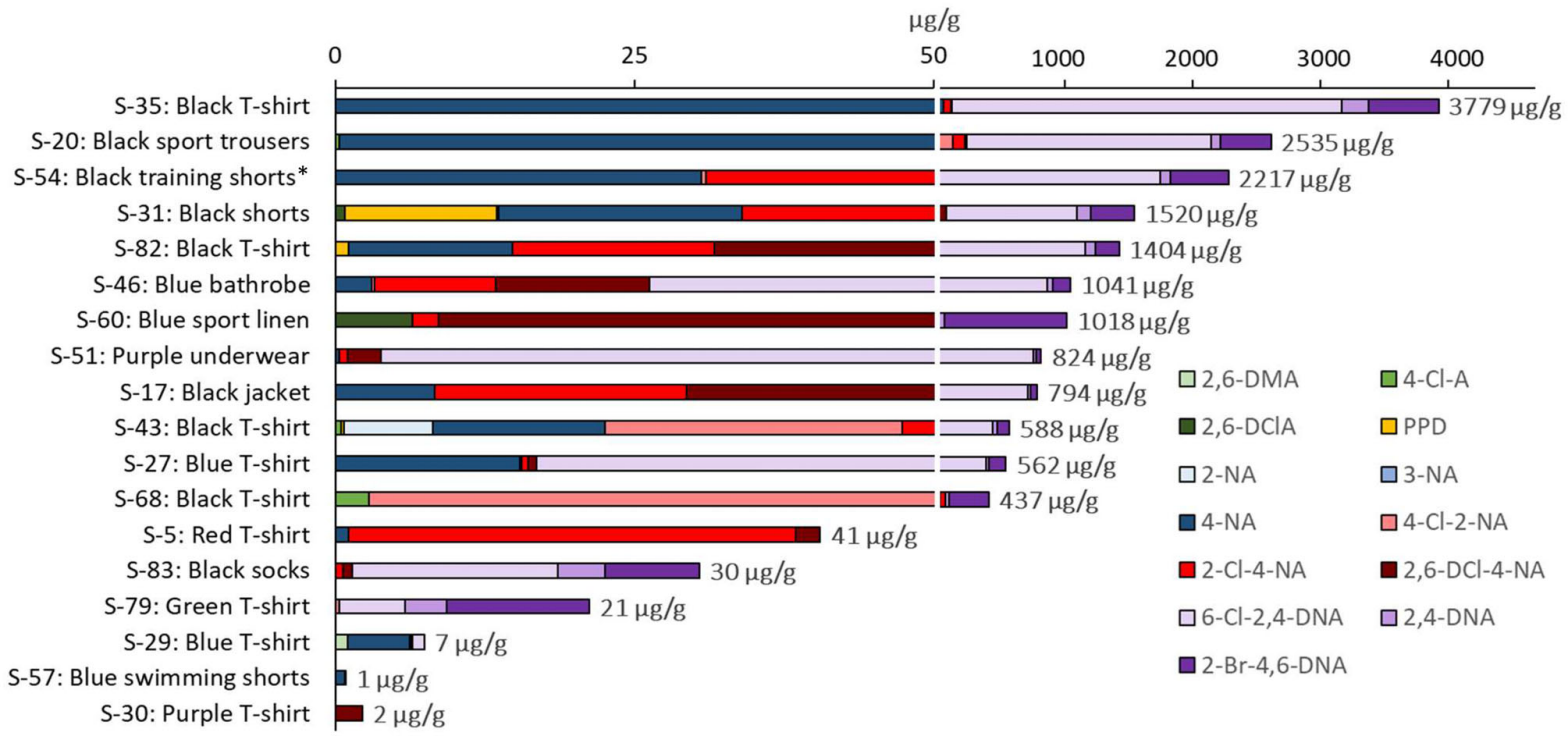
Quantified arylamines in selected samples (*n* = 18). Abbreviations within brackets: 2,6‐dimethylaniline (2,6‐DMA), 4‐chloroaniline (4‐Cl‐A), 2,6‐dichloroaniline (2,6‐DClA), PPD, 2‐nitroaniline (2‐NA), 3‐nitroaniline (3‐NA), 4‐nitroaniline (4‐NA), 4‐chloro‐2‐nitroaniline (4‐Cl‐2‐NA), 2‐chloro‐4‐nitroaniline (2‐Cl‐4‐NA), 2,6‐dichloro‐4‐nitroaniline (2,6‐DCl‐4‐NA), 6‐chloro‐2,4‐dinitroaniline (6‐Cl‐2,4‐DNA), 2,4‐dinitroaniline (2,4‐DNA), 2‐bromo‐4,6‐dinitroaniline (2‐Br‐4,6‐DNA). * made of 100% recycled polyester. A complete list of the investigated garments with sample numbers (S‐X) and textile materials are given in Table S2. The detection frequencies and levels of arylamines can be found in Table 2

**FIGURE 7:**
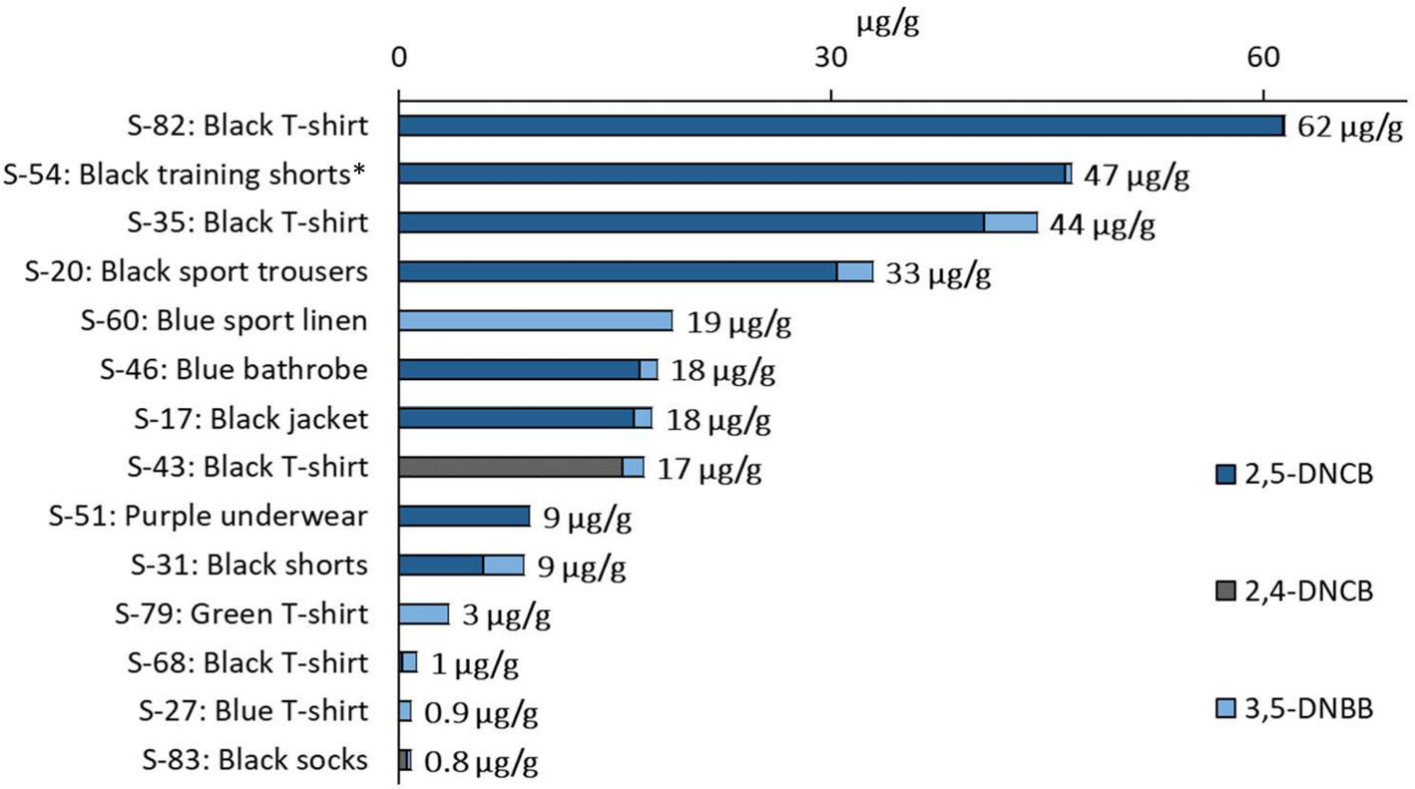
Quantified halogenated dinitrobenzenes in selected samples (*n* = 14). Abbreviations within brackets: 1‐chloro‐2,5‐dinitrobenzene (2,5‐DNCB), 1‐chloro‐2,4‐dinitrobenzene (2,4‐DNCB), 1‐bromo‐3,5‐dinitrobenzene (3,5‐DNBB). * made of 100% recycled polyester. A complete list of the investigated garments with sample numbers (S‐X) and textile materials are given in Table S2. The detection frequencies and levels can be found in Table 2

Apart from DDs, the present screening was able to identify other dye components in the garments, such as 13 different arylamines (Table 2). Eight were found to occur at a frequency higher than 10% and are shown in Figure 5. Except for 4‐chloroaniline (4‐Cl‐A), neither of these substances is regulated by REACH. Arylamines may be formed by decomposition, that is, reductive cleavage of the azo DDs. However, the azo DDs were found to be stable in the analytical procedure and not form arylamines as artefacts. Thus, it could be concluded that the identified arylamines were present in the garments as free compounds. For two of these compounds, 6‐chloro‐2,4‐dinitroaniline (6‐Cl‐2,4‐DNA) and 2‐bromo‐4,6‐dinitroaniline (2‐Br‐4,6‐DNA), the individual concentrations reached up to 3 mg/g. The garments with the highest total levels were all of black colours, as shown in Figure 6. Garments of recycled polyester were also investigated, and among these, a pair of black training shorts (S‐54) contained 2 mg/g of arylamines. It was also possible to identify halogenated dinitrobenzene compounds (Table 2, Figure 5), including the extreme skin sensitizer 1‐chloro‐2,4‐dinitrobenzene (2,4‐DNCB). As for the arylamines, the highest total levels were detected in black garments (Figure 7). Among these compounds was 2,4‐DNCB, detected in 6% of the selected garments, while 2,5‐DNCB and 1‐bromo‐3,5‐dinitrobenzene (3,5‐DNBB) were detected in 18% and 24%, respectively, of the garments.

**TABLE 2:** Arylamines and halogenated dinitrobenzene compounds quantified in 82 synthetic garments

Compound	Abbrev.	Frequency (%)	Concentration (μg/g)			
			Min	Max	Mean	Median
Arylamines						
4‐Nitroaniline	4‐NA	44	0.013	200	15	3.0
6‐Chloro‐2,4‐dinitroaniline	6‐Cl‐2,4‐DNA	39	1.0	2900	440	39
2,6‐Dichloro‐4‐nitroaniline	2,6‐DCl‐4‐NA	37	0.12	94	14	2.2
2‐Chloro‐4‐nitroaniline	2‐Cl‐4‐NA	35	0.11	86	14	5.9
2‐Bromo‐4,6‐dinitroaniline	2‐Br‐4,6‐DNA	34	0.38	3000	290	110
2,4‐Dinitroaniline	2,4‐DNA	30	0.51	260	47	28
4‐Chloro‐2‐nitroaniline	4‐Cl‐2‐NA	22	0.15	600	49	1.7
4‐Chloroaniline	4‐Cl‐A	11	0.073	6.9	1.4	0.48
1,4‐Phenylenediamine	PPD	9	0.26	27	7.5	1.6
2‐Nitroaniline	2‐NA	7	0.18	48	12	5.5
2,6‐Dichloro‐1,4‐phenylenediamine	2,6‐DCl‐PPD	7	0.58	6.5	2.5	0.83
3‐Nitroaniline	3‐NA	4	0.041	0.15	0.082	0.055
2,6‐Dimethylaniline	2,6‐DMA	1	1.0	1.0	–	–
Halogenated dinitrobenzene compounds						
1‐Bromo‐3,5‐dinitrobenzene	3,5‐DNBB	24	0.15	76	7.6	1.5
1‐Chloro‐2,5‐dinitrobenzene	2,5‐DNCB	18	0.17	61	18	9.1
1‐Chloro‐2,4‐dinitrobenzene	2,4‐DNCB	6	0.48	15	3.8	0.93
1‐Chloro‐2,6‐dinitrobenzene	2,6‐DNCB	1	1.6	1.6	–	–

## 4 DISCUSSION

A recent study was able to identify 12 different azo DDs in children's sports clothes purchased on the US market.^12^ The majority of these dyes were also identified in the present study together with more than 50 other azo DDs. Further, our results suggest that dyestuffs used in the manufacturing process contain not only a complex mixture of DDs but also a variety of impurities and dye precursors. Nitroanilines, halogenated nitroanilines and halogenated dinitrobenzene compounds are most likely introduced already in the dyeing process and then remain in the finished garments. To the best of our knowledge, this is the first time halogenated dinitrobenzene compounds, such as 2,4‐DNCB, have been identified in garments from the open market. Apart from the well‐known skin sensitizer 2,4‐DNCB, several of the other detected non‐regulated chemicals might be skin sensitizing and play an important role in textile allergy.

Arylamines were detected as free compounds at high levels in the textiles, while azo DDs generally occurred at much lower levels. The total concentration of arylamines exceeded 1 mg/g in seven of the garments. For example, in the black T‐shirt S‐82 the total level of arylamines were approximately 3500 times higher than the concentration of the identified azo DDs (Figures 4 and 6). This suggests that arylamines in this apparel constitute a significantly higher health risk than the content of azo DDs. The highest concentrations for some arylamines, such as halogenated dinitroanilines, reached 3 mg/g, which is 100 times higher than the EU limits for regulated arylamines, that is, 30 μg/g. Halogenated dinitroanilines were found to be frequently occurring, in total detected in about 50% of the garments. Interestingly, the black training shorts made of 100% recycled polyester, were also shown to contain high levels of arylamines. This emphasizes the need for a more extensive chemical control of the recycled textiles used as starting materials in the production of new textile fibres.

To summarize, exposure to other textile dyes and dye components seems to be more common than to the eight TDM dyes in the EBS, which is in agreement with previous findings.^11^ Two of these dyes could not be detected in any of the investigated textile samples, two had a detection frequency <5%, and the rest <10% except for DO3 which was detected in 26% of the samples. Furthermore, we have shown the occurrence of other potential skin‐sensitizing dye components in the investigated garments at a high frequency, and occasionally very high concentrations. This implies two things. Firstly: The composition of dyes in the EBS is not representative of the dyes occurring in garments on the common market. Also, the content of dye components in the dyes used for patch testing has to be taken into consideration. Secondly, the frequent occurrence of arylamines, such as nitroanilines and halogenated dinitroanilines, incomparably much higher concentrations than the dyes in the garments, makes these arylamines possible candidates as culprits in skin sensitization and allergic reactions. Part of the skin reactions commonly reported from patch testing with TDM might be due to cross‐reactions with one or several of these substances detected in the present study. According to the literature, it is not uncommon to observe cross‐reactions among azo dyes and *p*‐amino‐substituted compounds in already sensitized individuals.^30^

This study has revealed that there are complex mixtures of chemicals present in garments from the open market. This is demonstrated in Figures 2A and 3, where the latter shows the total ion chromatogram (TIC) from a GC/MS analysis in which several peaks have been assigned to arylamines and other aromatic compounds. As can be seen, there are also several large peaks in the later part of the chromatogram representing compounds that so far not have been identified. Since this is a full scan MS using standard EI, the relative amount of each compound is roughly given by the peak areas. Thus, there is a number of unidentified compounds in this textile sample present in the same, or even higher, concentration range as the identified compounds. In Figure 2A, the chromatogram of the LC/MS analysis is shown where DDs are determined, here with the example of Disperse Red 167:1. The TIC displayed in grey demonstrates the complex composition of the sample extract. It indicates the presence of more than 80 peaks, each representing one or more compounds. Since the ionization efficiency and thus response in HPLC/MS varies strongly between different compounds, it is not possible to estimate the amount in the unknown peaks.

## 5 CONCLUSION

The present study shows that synthetic skin‐close garments from the Swedish open market contain a large variety of azo DDs, arylamines and halogenated dinitrobenzene compounds. Generally, arylamines were found to occur in the garments at much higher levels as compared to azo dyes, thus most likely constituting a higher health risk. As these are free compounds and non‐covalently bound to the textile fibres, the arylamines can migrate to the skin. Many of the chemicals identified in this study are not regulated today but could be possible health hazards and relevant for textile allergies. Thus, these substances should be further investigated in terms of migration from clothes to the skin, skin uptake and sensitization potency. Furthermore, the efficiency of laundry should be investigated regarding the removal of azo dyes, arylamines and halogenated dinitrobenzenes. The presence of these chemicals in textiles is also an important aspect when using recycled textiles in the manufacturing of new textile fibres to achieve a sustainable circular economy. This study has shown the importance of analytical chemical screening methods for textiles to enable the identification and control of the content of possible skin sensitizers. Finally, the results demonstrate that complex mixtures of chemicals, not only azo DDs and arylamines, are present in textiles sold on the open market. This demonstrates that more research is needed to obtain a much better knowledge and understanding of the chemicals we are exposed to on a daily basis by wearing clothes.

## AUTHOR CONTRIBUTIONS

**Josefine Carlsson:** Investigation; writing – original draft; methodology; visualization; writing – review and editing; formal analysis; data curation. **Tim Åström:** Investigation; writing – original draft; writing – review and editing; visualization; methodology; formal analysis; data curation. **Conny Östman:** Conceptualization; funding acquisition; writing – original draft; writing – review and editing; supervision; resources. **Ulrika Nilsson:** Conceptualization; writing – original draft; writing – review and editing; project administration; supervision; resources; funding acquisition.

## CONFLICT OF INTEREST

The authors declare no conflict of interest.

## Supporting information


**Appendix S1** Supporting Information

## Data Availability

The data that supports the findings of this study are available in the supplementary material of this article
