# Supplementary material for: Disperse azo dyes, arylamines and halogenated dinitrobenzene compounds in synthetic garments on the Swedish market
Source: Contact Dermatitis. 2022 Jun 10;87(4):315–24. doi: 10.1111/cod.14163 (PMC9542814; doi:10.1111/cod.14163)
Supplement: Supplementary file 1 — Appendix S1 Supporting Information [file COD-87-315-s001.docx]

**Disperse azo dyes, arylamines and halogenated dinitrobenzene compounds in synthetic garments on the Swedish market**

Josefine Carlsson^†^, Tim Åström^†^, Conny Östman, Ulrika Nilsson

Department of Materials and Environmental Chemistry

Stockholm University, SE-106 91 Stockholm, Sweden

^†^ shared first authorship

#### **Detailed description of sample preparation and instrumental analysis**

#### **Sample preparation for HPLC/HRMS screening of disperse dyes (DDs)**

#### After ultrasonic extraction, a volume of 200 µL of ultrapure water was added to the pooled dichloromethane extract and the resulting volume reduced to 200 µL under a gentle flow of nitrogen at 35°C. A volume of 800 µL was then added, followed by syringe filtration (PTFE, 0.45µm, NTK Kemi, Uppsala, Sweden) into a vial. A volume of 5 µL was injected into the HPLC/HRMS.

#### **Sampling preparation for HPLC/TSQ MS of DDs**

The sample processing was performed as for the HPLC/HRMS screening, except that 10 µg each of surrogate internal standards (IS), quinoline-d_7_, 3-nitroaniline-d_4_ and 4-nitroaniline-^15^N_2,_ and 16.9 µg of Sudan-I-d_5_ were added prior to ultrasonic extraction. and the pooled extract volume was reduced to approx. 5 mL. Each sample was then subjected to filtration by using a syringe filter (RC, 0.2µm, Phenomenex, Torrance, CA, US) and the volume was further reduced to 1 mL, after which 5 µg of 1-indanone was added as volumetric internal standard. A volume of 500 µL of deionized water was added prior to and 500 µL of acetonitrile after evaporation of the extraction solvent. An additional syringe filtration step was carried out if necessary. An injection volume of 10 µL from each sample extract was subjected to HPLC/TSQ MS analysis for target screening and confirmation of disperse dyes. If necessary, to avoid matrix effects, the samples were diluted 5-10 times prior to injection on HPLC/TSQ MS.

#### **Sampling preparation for GC/MS of arylamines and halogenated dinitrobenzenes**

The sample processing was performed as for the HPLC/TSQ MS analysis, with addition of internal standards prior to ultrasonic extraction. The pooled dichloromethane extract was reduced to 1 mL, after which 5 µg of 1-indanone was added as volumetric internal standard. One µl of the reduced extract was injected on GC/MS.

#### **HPLC/HRMS screening of disperse dyes (DDs)**

A UHPLC/electrospray (ESI)-Orbitrap^TM^ high-resolution mass spectrometry (HRMS) instrument (Q Exactive HF, Thermo Scientific, Rockford, IL, USA) was used for the analyses. HPLC was performed using an Acquity UPLC^®^ BEH C_18_ column (50x2.1 mm, 1.7-µm particles), with a C_18_ guard column (Waters, Aberdeen, Scotland) connected in front. Mobile phase A consisted of ultrapure water with 10 mM of ammonium acetate, and mobile phase B was acetonitrile with 10% ultrapure water.

At a flow rate of 0.4 mL/min a linear gradient was applied starting with a composition of 5% B for one min and then ramped up during 20 min to the final composition of 95% B. The latter condition was kept for 5 min before returning to initial settings. Conditioning time prior to the next run was 2.5 min resulting in a total analysis time of 27.5 min.

The ESI settings in the positive mode were: sheath gas flow rate 25 (arbitrary units), auxiliary gas flow rate 7 (arbitrary units), spray voltage 4.1 kV, auxiliary gas temp 320°C, and auxiliary gas heater temp 240°C.

The MS acquisition was performed in full scan MS combined with data dependent MS^2^ (dd-MS2) with an inclusion list activated (**Table SI-5**). The inclusion list of suspected non-regulated azo DDs was based on published information regarding usage in industry^22^ and available reference standards. The full scan range was set to 66.7 – 1000 m/z with a resolution (m/Δm, FWHM) 120,000 at m/z 400, automatic gain control (AGC) target at 3x10^6^, and maximum injection time 256 ms. For dd-MS2, the resolution was set to 15,000 with AGC target at 10^5^ and maximum injection time 32 ms. The isolation window was set to 0.4 m/z and normalized collision energy (NCE) 50 eV.

We have recently published a data treatment workflow for suspect screening^29^. This methodology was also used for the present suspect screening of disperse dyes. Briefly, detection of the adduct ions [M+H]^+^, [M+NH_4_^]+^, [M+K]^+^, [M+Na]^+^ and [M+acetonitrile+H]^+^ was done by use of the Compound Discoverer 3.1 software (Thermo Scientific, Massachusetts). Obtained accurate mass and predicted molecular formulas were used for search in ChemSpider (Royal Society of Chemistry, London, England) within a 5-ppm mass accuracy. Compound Discoverer software matched the experimental obtained MS^2^ data with mzCloud data (HighChem LLC, Slovakia). Further, the inclusion list (**Table SI-5**) was used for assigning molecular formulas and names with a 5-ppm tolerance. The obtained data were extracted to a datafile in csv-format for further processing with Excel. Further filtering of data was performed by comparision of experimental HPLC retention times to those predicted from logP (**SI-6**).

#### **HPLC/TSQ MS of DDs**

To confirm and quantify the dyes identified by the HPLC/HRMS screening, target analysis with HPLC/triple-stage quadrupole (TSQ) MS and standard compounds was performed with a Waters Acquity I-Class UPLC system (Waters, Manchester, UK) coupled with a Xevo TQ-S instrument equipped with an ESI source (Waters, Manchester, UK). The LC column was a Kinetex EVO C18 column (150 mm x 2.1 mm, 2.6-µm particles) from Phenomenex. A gradient system was used, with solvent A being 10% acetonitrile (aq) and B 90% acetonitrile (aq), both containing 10mM ammonium acetate. The column oven temperature was 40°C and the flow rate was set to 0.4 mL/min. The gradient program was as follows: 0-2 min 100% A; 2-8.5 min 100%-50% A; 8.5-15 min isocratic with 50% A; 15-16 min 50-25% A; 16-18.5 min 25% A; 19-21 min 0% A. The system was equilibrated at initial conditions for two min before next injection.

HPLC/TSQ MS in multiple-reaction mode (MRM) was performed in both positive ESI at a capillary voltage of 0.5 kV, and in negative ESI at -2.5 kV. The two most intense daughter ions were selected for each dye. For all compounds, the source temp was 150°C, desolvation temp 650°C, cone gas flow 80 mL/min and desolvation gas flow 850 mL/min. Details about the MRM settings and transitions are given in **Table SI-7**.

The method limit of quantification (S/N=10) was determined to 0.7 – 20.5 ng/g of textile, depending on the dye. No matrix effects could be observed and the instrumental response was linear (R^2^>0.99) within the entire measured range of 0.02 to 1.6 ng amount injected. The relative standard deviation was within the range 1 – 13% (n=3).

The extraction recoveries were evaluated using a triplicate of a spiked dye-free sample, in this case a white sock made of 100% polyester. The recoveries normalised to IS were between 85% and 114%, except for Sudan IV which had a yield of approx. 50% from the textile, see **Table SI-3**.

#### **GC/MS of arylamines**

For detection of arylamines in the garments, an Agilent 5975C MSD equipped with a 6890N GC, a 7693 autosampler, and a PTV-injector (Agilent Technologies, Palo Alto, CA, USA) was used. The GC column was a CP-Sil 8 CB (30 m, Ø = 0.25 m, d_f_ = 0.25 µm, Agilent Technologies). The GC injection temperature was held at 70°C for 0.05 min, and was then ramped at 700°C/min to 300°C, with the final setting held for 8 min. The GC oven program started at 50°C and was immediately ramped at 6°C/min to 165°C, which was held for 3.5 min, followed by a 15°C/min ramp to 250°C, held for 1.5 min. A third ramp of 15°C/min to a final temperature of 325°C was then applied, with the final setting held for 13.5 min. The total run time was 48.3 min. The MS analyses were performed in electron ionization mode at 70eV, with the ion source operating at 250°C. The temperature of the quadrupole mass analyzer was set to 200°C. The MS acquisition was performed in full scan mode monitoring m/z 45-570. For identification and quantification, three ions were used for each compound, one quantifier ion and two qualifier ions, to compare the ion ratios and retention times in samples with standards, see **Table SI-8**.

The method quantification limit for amines (S/N=10) was determined to 9.9 - 319.2 ng/g of textile, depending on compound. The corrected recoveries for spiked matrix were in the range 83 - 106 %, except for 1,4-phenylenediamine (28%) and 2,6-dichlorobenzene-1,4-diamine (66%), **Table SI-4.** No matrix effects could be observed and the instrumental response was linear, with coefficients of determination (R^2^) in the range 0.957 - 0.996 for the entire measured range 0.4 - 9.6 ng amount injected, except for PPD. This compound suffered from chromatographic peak distorsion with poor linearity as a result. The relative standard deviation for triplicate samples was in the range 1 - 7%, except for PPD (22%).

**SI-1 – Analytical standards**

A.S. = Analytical standard, NS = Not specified

| **Name** | **CAS #** | **Abbreviation** | **Molecular formula** | **Purity** | **Supplier** |
| --- | --- | --- | --- | --- | --- |
| **Dyes** | | | | | |
| Disperse Orange 3 | 730-40-5 | DO3 | C_12_H_10_N_4_O_2_ | 90% | Sigma Aldrich, St Louis, USA |
| Disperse Yellow 3 | 2832-40-8 | DY3 | C_15_H_15_N_3_O_2_ | 30% | Sigma Aldrich, St Louis, USA |
| Disperse Orange 1 | 2581-69-3 | DO1 | C_18_H_14_N4O_2_ | A.S. | Sigma Aldrich, St Louis, USA |
| Disperse Orange 37 | 13301-61-6 | DO37 | C_17_H_15_Cl_2_N_5_O_2_ | A.S. | Sigma Aldrich, St Louis, USA |
| Disperse Orange 13 | 6253-10-07 | DO13 | C_22_H_16_N_4_O | 90% | Sigma Aldrich, St Louis, USA |
| Disperse Orange 25 | 31482-56-1 | DO25 | C_17_H_17_N_5_O_2_ | 90% | Sigma Aldrich, St Louis, USA |
| Disperse Blue 14 | 2475-44-7 | DB14 | C_16_H_14_N_2_O_2_ | 97% | Sigma Aldrich, St Louis, USA |
| Disperse Red 1 | 2872-52-8 | DR1 | C_16_H_18_N_4_O_3_ | 95% | Sigma Aldrich, St Louis, USA |
| Disperse Red 13 | 3180-81-2 | DR13 | C_16_H_17_ClN_4_O_3_ | 95% | Sigma Aldrich, St Louis, USA |
| Disperse Blue 124 | 61951-51-7 | DB124 | C_16_H_19_N_5_O_4_S | Microscopy | Sigma Aldrich, St Louis, USA |
| Disperse Blue 35 | 12222-75-2 | DB35 | C_15_H_12_N_2_O_4_ | Microscopy | Sigma Aldrich, St Louis, USA |
| Disperse Blue 106 | 12223-01-7 | DB106 | C_14_H_17_N_5_O_3_S | NS | Chemotechnique, Vellinge, Sweden |
| Disperse Red 17 | 3179-89-3 | DR17 | C_17_H_20_N_4_O_4_ | A.S. | Sigma Aldrich, St Louis, USA |
| Sudan IV | 85-83-6 | SRIV | C_24_H_20_N_4_O | ≥80% | Sigma Aldrich, St Louis, USA |
| Sudan I | 842-07-9 | SRI | C_16_H_12_N_2_O | >95% | Sigma Aldrich, St Louis, USA |
| Disperse Black 2 | 6232-57-1 | DB2 | C_14_H_16_N_4_O | NS | Sigma Aldrich, St Louis, USA |
| Disperse Blue 337 | 65916-12-3 | DB337 | C_24_H_27_N_7_O_5_ | NS | Sigma Aldrich, St Louis, USA |
| Disperse Blue 79 | 12239-34-8 | DB79 | C_24_H_27_BrN_6_O_10_ | NS | Sigma Aldrich, St Louis, USA |
| Disperse Brown 22 | 63467-07-2 | DB22 | C_16_H_16_Cl_2_N_4_O_4_ | NS | Sigma Aldrich, St Louis, USA |
| Disperse Red 5 | 3769-57-1 | DR5 | C_17_H_19_ClN_4_O_4_ | NS | Sigma Aldrich, St Louis, USA |
| Disperse Red 60 | 17418-58-5 | DR60 | C_20_H_13_NO_4_ | NS | Sigma Aldrich, St Louis, USA |
| Methyl Orange | 547-58-0 | MO | C_14_H_14_N_3_NaO_3_S | NS | BDH Chemicals Ltd, Poole, England |
| Disperse Red 19 | 2734-52-3 | DR19 | C_16_H_18_N_4_O_4_ | 97% | Sigma Aldrich, St Louis, USA |
| Sudan I-d_5_ (IS) | 752211-63-5 | SRI-d5 | C_16_D_5_H_7_N_2_O | A.S. | Sigma Aldrich, St Louis, USA |
| **Arylamines** | | | | | |
| 4-chloroaniline | 106-47-8 | 4-Cl-A | C_6_H_6_ClN | 98% | Sigma Aldrich, St Louis, USA |
| 2-bromoaniline | 615-36-1 | 2-Br-A | C_6_H_6_BrN | 98% | Sigma Aldrich, St Louis, USA |
| 3-bromoaniline | 591-19-5 | 3-Br-A | C_6_H_6_BrN | 98% | Sigma Aldrich, St Louis, USA |
| 4-bromoaniline | 106-40-1 | 4-Br-A | C_6_H_6_BrN | 97% | Sigma Aldrich, St Louis, USA |
| 2,6-dimethylaniline | 87-62-7 | 2,6-DMA | C_8_H_11_N | 98% | Fluka Chemicals, Germany |
| *p*-anisidine | 104-94-9 | p-Ani | C_7_H_9_NO | 99% | Sigma Aldrich, St Louis, USA |
| 1,4-phenylenediamine | 106-50-3 | PPD | C_6_H_8_N_2_ | NS | Sigma Aldrich, St Louis, USA |
| 2-amino-p-cresol | 95-84-1 | Cresol | C_7_H_9_NO | 97% | Sigma Aldrich, St Louis, USA |
| 2,6-dichloro-1,4-phenylenediamine | 609-20-1 | 2,6-DCl-PPD | C_6_H_6_Cl_2_N_2_ | 96% | Sigma Aldrich, St Louis, USA |
| 2,6-dichloroaniline | 608-31-1 | 2,6-DClA | C_6_H_5_Cl_2_N | 96% | Sigma Aldrich, St Louis, USA |
| Diphenylamine | 122-39-4 | Di-ph-A | C_12_H_11_N | A.S. | Sigma Aldrich, St Louis, USA |
| 2-nitroaniline | 88-74-4 | 2-NA | C_6_H_6_N_2_O_2_ | 98% | Sigma Aldrich, St Louis, USA |
| 3-nitroaniline | 99-09-2 | 3-NA | C_6_H_6_N_2_O_2_ | 98% | Sigma Aldrich, St Louis, USA |
| 4-nitroaniline | 100-01-6 | 4-NA | C_6_H_6_N_2_O_2_ | ≥99% | Sigma Aldrich, St Louis, USA |
| 5-chloro-2-nitroaniline | 1635-61-6 | 5-Cl-2-NA | C_6_H_5_ClN_2_O_2_ | 97% | Sigma Aldrich, St Louis, USA |
| 4-chloro-2-nitroaniline | 89-63-4 | 4-Cl-2-NA | C_6_H_5_ClN_2_O_2_ | 99% | Sigma Aldrich, St Louis, USA |
| 2-chloro-4-nitroaniline | 121-87-9 | 2-Cl-4-NA | C_6_H_5_ClN_2_O_2_ | 99% | Sigma Aldrich, St Louis, USA |
| 2,4-dinitroaniline | 97-02-9 | 2,4-DNA | C_6_H_5_N_3_O_4_ | 98% | Sigma Aldrich, St Louis, USA |
| 2,6-dichloro-4-nitroaniline | 99-30-9 | 2,6-DCl-4-NA | C_6_H_4_Cl_2_N_2_O_2_ | 96% | Sigma Aldrich, St Louis, USA |
| 6-chloro-2,4-dinitroaniline | 3531-19-9 | 6-Cl-2,4-DNA | C_6_H_4_ClN_3_O_4_ | 97% | Sigma Aldrich, St Louis, USA |
| 2-bromo-4,6-dinitroaniline | 1817-73-8 | 2-Br-4,6-DNA | C_6_H_4_BrN_3_O_4_ | 94% | Sigma Aldrich, St Louis, USA |
| **Halogenated dinitrobenzene compounds** | | | | | |
| 1-chloro-2,4-dinitrobenzene | 97-00-7 | 2,4-DNCB | C_6_H_3_ClN_2_O_4_ | 97% | Sigma Aldrich, St Louis, USA |
| 1-chloro-2,5-dinitrobenzene | 619-16-9 | 2,5-DNCB | C_6_H_3_ClN_2_O_4_ | NS | Toronto Research Chemicals, Toronto, CA |
| 1-chloro-2,6-dinitrobenzene | 606-21-3 | 2,6-DNCB | C_6_H_3_ClN_2_O_4_ | NS | Toronto Research Chemicals, Toronto, CA |
| 1-chloro-3,4-dinitrobenzene | 610-40-2 | 3,4-DNCB | C_6_H_3_ClN_2_O_4_ | 90% | Alfa Aesar, Kandel Germany |
| 1-bromo-3,5-dinitrobenzene | 18242-39-2 | 3,5-DNBB | C_6_H_3_BrN_2_O_4_ | NS | Apollo Scientific, Stockport, UK |

### **SI-2 – Investigated garments**

Sample # refers to sample number (S-X in Figure 4, 6, 7).

| **Sample #** | **Color** | **Sort** | **Fiber composition** | | **Country** |
| --- | --- | --- | --- | --- | --- |
|  |  |  | **Polyester [%]** | **Other [%]** |  |
| 1 | Pink | Top | 88 | 12% elastane | Unknown |
| 2 | Blue | Trousers | 100 |  | China |
| 3 | Pink | Top | 88 | 12% elastane | Sri Lanka |
| 4 | Pink | T-shirt | 100 |  | Vietnam |
| 5 | Red | T-shirt | 100 |  | Unknown |
| 6 | Yellow | T-shirt | 100 |  | Cambodia |
| 7 | Blue | T-shirt | 100 |  | Philippines |
| 8 | Red | T-shirt | 100 |  | Georgia |
| 9 | Blue | T-shirt | 100 |  | Indonesia |
| 10 | Yellow | T-shirt | 100 |  | Bangladesh |
| 11 | Yellow | T-shirt | 100 |  | China |
| 12 | Green | T-shirt | 87% recycled | 13% elastane | China |
| 13 | Blue | T-shirt | 90 | 10% elastane | China |
| 14 | Blue | Trousers | 100 |  | China |
| 15 | Orange | Skirt | 87 | 13% elastane | Indonesia |
| 16 | Black | Shorts | 88 | 12% elastane | Vietnam |
| 17 | Black | Jacket | 100 |  | China |
| 18 | Purple | T-shirt | 100% recycled |  | Thailand |
| 19 | Blue | T-shirt | 100 |  | Cambodia |
| 20 | Black | Sport trousers | 100 |  | China |
| 21 | Orange | T-shirt | 100 |  | Thailand |
| 22 | White | Socks | 70 | 25% cotton 5% elastane | Unknown |
| 23 | Pink | T-shirt | 100 |  | China |
| 24 | Pink | T-shirt | 100 |  | Bangladesh |
| 25 | Blue | Dress | 85 | 15% elastane | Cambodia |
| 26 | Pink | T-shirt | 100 |  | China |
| 27 | Blue | T-shirt | 90 | 10% elastane | Unknown |
| 28 | Blue | T-shirt | 100 |  | Unknown |
| 29 | Blue | T-shirt | 91 | 9% elastane | Indonesia |
| 30 | Purple | T-shirt | 88 | 12% elastane | Unknown |
| 31 | Black | Shorts | 100 |  | China |
| 32 | Pink | T-shirt | 95 | 5% elastane | Unknown |
| 33 | Blue | T-shirt | 100 |  | Indonesia |
| 34 | Black | T-shirt | 100 |  | Vietnam |
| 35 | Black | T-shirt | 100 |  | India |
| 36 | Black | Socks | 70 | 25% cotton 5% elastane | Unknown |
| 37 | Blue | T-shirt | 100 |  | Unknown |
| 38 | Pink | Swimsuit | 100 |  | Indonesia |
| 39 | Yellow | T-shirt | 100 |  | Indonesia |
| 40 | Blue | T-shirt | 85 | 15% elastane | China |
| 41 | Black | T-shirt | 100 |  | Vietnam |
| 42 | Red | T-shirt | 85 | 15% cotton | China |
| 43 | Black | T-shirt | 100 |  | China |
| 44 | Red | T-shirt | 87 | 13% elastane | China |
| 45 | Pink | Bra | 100 |  | China |
| 46 | Blue | Bathrobe | 100 |  | Unknown |
| 47 | Blue | Bathrobe | 100 |  | China |
| 48 | White | Shorts | 100 |  | China |
| 49 | Yellow | Underwear | 90 | 10%elastane | Bangladesh |
| 50 | Red | Underwear | 90 | 10% elastane | Bangladesh |
| 51 | Purple | Underwear | 90 | 10% elastane | Bangladesh |
| 52 | Black | T-shirt | 100% recycled |  | Bangladesh |
| 53 | Yellow | T-shirt | 100% recycled |  | Bangladesh |
| 54 | Black | Training shorts | 100% recycled |  | Bangladesh |
| 55 | Red | Jumpsuit | 100 |  | Unknown |
| 56 | Red | Dress | 99 | 1% elastane | China |
| 57 | Blue | Swimming shorts | 93 | 7% elastane | China |
| 58 | Blue | Top | 86 | 14% elastane | China |
| 59 | Blue | UV-overall | 82 | 18% elastane | China |
| 60 | Blue | Sport linen | 100 |  | China |
| 61 | Orange | T-shirt | 92 | 8% elastane | Bangladesh |
| 62 | Pink | Swimsuit | 90 | 10% elastane | China |
| 63 | Black | Underskirt | 100 |  | Bangladesh |
| 64 | Yellow | T-shirt | 100 |  | Bangladesh |
| 65 | Blue | T-shirt | 100 |  | Bangladesh |
| 66 | Blue | T-shirt | 100 |  | Bangladesh |
| 67 | Green | T-shirt | 100 |  | Bangladesh |
| 68 | Black | T-shirt | 72 | 24% viscose, 4% elastane | Bangladesh |
| 69 | Green | T-shirt | 100 |  | Bangladesh |
| 70 | Red | Sweater | 97 | 3% metal fiber | China |
| 71 | Dark Red | T-shirt | 88 | 12% elastane | Bangladesh |
| 72 | Orange | T-shirt | 100% recycled |  | Cambodia |
| 73 | Green | T-shirt | 92 | 8% elastane | Cambodia |
| 74 | Orange | T-shirt | 100 |  | Cambodia |
| 75 | Blue | T-shirt | 90 | 10% elastane | Bangladesh |
| 76 | Yellow | T-shirt | 96 | 4% elastane | China |
| 77 | Yellow/Green | T-shirt | 92 | 8% elastane | Cambodia |
| 78 | Green | T-shirt | 88 | 12% elastane | Bangladesh |
| 79 | Green | T-shirt | 100 |  | Cambodia |
| 80 | Green | T-shirt | 95 | 5% cotton | Bangladesh |
| 81 | Blue | Shorts | 100 |  | China |
| 82 | Black | T-shirt | 100 |  | Cambodia |
| 83 | Black | Socks | 100 |  | China |

### **SI-3 - Matrix-spiked normalized recoveries of disperse dyes**

Mean ± standard deviation, and relative standard deviation (CV) for recovery experiments of 22 selected dyes. The recoveries are normalized against IS Sudan I-d_5_

| **Compound** | **Mean ± std**  **[%]** | **CV**  **[%]** |
| --- | --- | --- |
| Disperse Red 19 | 107.2 ± 3.9 | 4 |
| Disperse Black 2 | 94.8 ± 3.1 | 3 |
| Disperse Red 17 | 107.4 ± 5.6 | 5 |
| Disperse Yellow 3 | 105.6 ± 3.6 | 3 |
| Disperse Brown 22 | 105.6 ± 5.9 | 6 |
| Disperse Blue 106 | 101.2 ± 3.1 | 3 |
| Disperse Orange 3 | 114.4 ± 4.2 | 4 |
| Disperse Red 5 | 108.5 ± 3.6 | 3 |
| Disperse Blue 14 | 97.7 ± 0.6 | 1 |
| Disperse Blue 35 | 96 ± 1.6 | 2 |
| Disperse Red 1 | 112.2 ± 4.6 | 4 |
| Disperse Blue 124 | 109.9 ± 4.6 | 4 |
| Disperse Orange 25 | 103.3 ± 4.4 | 4 |
| Sudan I | 94.4 ± 3.2 | 3 |
| Disperse Red 60 | 93 ± 5.6 | 6 |
| Disperse Blue 337 | 96.4 ± 3.3 | 3 |
| Disperse Orange 37 | 101.2 ± 4.2 | 4 |
| Disperse Orange 1 | 111.7 ± 3.9 | 3 |
| Disperse Blue 79 | 108.1 ±5.7 | 5 |
| Disperse Orange 13 | 85 ± 2.3 | 3 |
| Sudan IV | 47.7 ± 6.2 | 13 |
| Disperse Red 13 | 110.9 ± 4.2 | 4 |

### **SI-4 –Matrix-spiked recoveries of arylamines and halogenated dinitrobenzenes from the analytical procedure**

Mean ± standard deviation, and relative standard deviation (CV) for recovery experiments. Both absolute recoveries and recoveries normalized against IS (quinoline-d_7_, 3-nitroaniline-d_4_, 4-nitroaniline-^15^N_2_)

| **Compound** | **Absolute recovery [%]** | | **Normalized recovery [%]** | |
| --- | --- | --- | --- | --- |
|  | **Mean ± std** | **CV** | **Mean ± std** | **CV** |
| 4-chloroaniline | 73.5 ± 1.7 | 2 | 96 ± 2.4 | 2 |
| 1,4-phenylenediamine | 21.4 ± 4.3 | 20 | 28 ± 6.2 | 22 |
| 2-nitroaniline | 81.3 ± 2.2 | 3 | 106.1 ± 1.6 | 2 |
| 3-nitroaniline | 71.8 ± 2.3 | 3 | 84.3 ± 0.9 | 1 |
| 1-chloro-3,4-dinitrobenzene | 75.1 ± 2.1 | 3 | 88.1 ± 0.9 | 1 |
| 1-chloro-2,4-dinitrobenzene | 75.8 ± 3.5 | 5 | 88.8 ± 1.8 | 2 |
| 2,6-dichloro-1,4-phenylenediamine | 56 ± 3 | 5 | 65.7 ± 3.5 | 5 |
| 5-chloro-2-nitroaniline | 77 ± 1.7 | 2 | 90.3 ± 1.4 | 2 |
| 1-bromo-3,5-dinitrobenzene | 74.5 ± 2.5 | 3 | 87.3 ± 0.6 | 1 |
| 4-nitroaniline | 72.6 ± 1.7 | 2 | 85.2 ± 1 | 1 |
| 4-chloro-2-nitroaniline | 71 ± 1.3 | 2 | 83.3 ± 1.4 | 2 |
| 2-chloro-4-nitroaniline | 83.2 ± 3.3 | 4 | 97.5 ± 1.8 | 2 |
| 2.6-dichloro-4-nitroaniline | 78.7 ± 3.7 | 5 | 92.3 ± 2.4 | 3 |
| 6-chloro-2,4-dinitroaniline | 90.9 ± 3 | 3 | 106.6 ± 1.8 | 2 |
| 2,4-dinitroaniline | 86.4 ± 2.1 | 2 | 101.4 ± 2.1 | 2 |
| 2-bromo-4,6-dinitroaniline | 83.4 ± 3.2 | 4 | 97.8 ± 3.4 | 4 |

### **SI-5 – Inclusion list for suspect screening with HPLC/HRMS**

Exact m/z for [M+H]^+^ of selected analytes. The charge state is +1 for all analytes. Colorants marked with ^†^ contains more than one component.

| **Ion**  **[m/z]** | **Molecular formula** | **Compound name** |
| --- | --- | --- |
| 120.05562 | C_6_H_5_N_3_ | 1-H-benzotriazole |
| 128.02615 | C_6_H_6_ClN | 4-chloroaniline |
| 130.06513 | C_9_H_7_N | quinoline |
| 134.07127 | C_7_H_7_N_3_ | methyl-1H-benzotriazole |
| 136.02155 | C_7_H_5_NS | benzothiazole |
| 138.11689 | C_9_HD_7_N | quinoline-d_7_ |
| 139.0502 | C_6_H_6_N_2_O_2_ | 4-nitroaniline |
| 144.08078 | C_10_H_9_N | 2-methylquinoline |
| 148.08692 | C_8_H_9_N_3_ | 5,6-dimethyl-2H-benzotriazole |
| 158.09643 | C_11_H_11_N | 2,6-dimethylquinoline |
| 173.01123 | C_6_H_5_ClN_2_O_2_ | 4-chloro-2-nitroaniline |
| 182.00927 | C_8_H_7_NS_2_ | 2-methylthiobenzothiazole |
| 184.03528 | C_6_H_5_N_3_O_4_ | 2,4-dinitroaniline |
| 195.12557 | C_12_H_2_D_8_O_2_ | 4,4-dihydroxybiphenyl-d_8_ |
| 206.97226 | C_6_H_4_Cl_2_N_2_O_2_ | dichloran |
| 217.99631 | C_6_H_4_ClN_3_O_4_ | 2-chloro-4,6-dinitroaniline |
| 226.09749 | C_13_H_11_N_3_O | 2-(benzotriazol-2-yl)-4-methylphenol |
| 243.08765 | C_12_H_10_N_4_O_2_ | Disperse Orange 3 |
| 249.10224 | C_16_H_12_N_2_O | Sudan 1 |
| 254.13362 | C_16_D_5_H_7_N_2_O | Sudan 1-d_5_ |
| 257.13969 | C_14_H_16_N_4_O | Disperse Black 2 |
| 261.94579 | C_6_H_4_BrN_3_O_4_ | 2-bromo-4,6-dinitroaniline |
| 263.12912 | C_16_H_14_N_4_ | Disperse Black 1 |
| 265.08277 | C_13_H_16_N_2_S_2_ | N-cyclohexyl-2-benzothiazolesulphenamide |
| 266.0924 | C_15_H_11_N_3_O_2_ | Disperse Yellow 4 |
| 267.1128 | C_16_H_14_N_2_O_2_ | Disperse Blue 14 |
| 270.1237 | C_15_H_15_N_3_O_2_ | Disperse Yellow 3 |
| 285.08698 | C_15_H_12_N_2_O_4_ | Disperse Blue 35 |
| 300.10912 | C_14_H_13_N_5_O_3_ | Disperse Orange 1.1 |
| 301.12952 | C_15_H_16_N_4_O_3_ | Disperse Red 41 |
| 315.14517 | C_16_H_18_N_4_O_3_ | Disperse Red 1 |
| 319.11895 | C_18_H_14_N_4_O_2_ | Disperse Orange 1 |
| 324.1455 | C_17_H_17_N_5_O_2_ | Disperse Orange 25 |
| 325.09313 | C_16_H_12_N_4_O_4_ | Disperse Yellow 5 |
| 328.10403 | C_15_H_13_N_5_O_4_ | Disperse Yellow 119 |
| 331.14008 | C_16_H_18_N_4_O_4_ | Disperse Red 19 |
| 332.13935 | C_20_H_17_N_3_O_2_ | Disperse Yellow 27 |
| 332.18697 | C_20_H_21_N_5_ | Disperse Red Dye |
| 332.96431 | C_14_H_8_N_2_S_4_ | 2,2'-dithiobisbenzothiazole |
| 336.11249 | C_14_H_17_N_5_O_3_S | Disperse Blue 106 |
| 337.02536 | C_14_H_10_Cl_2_N_4_O_2_ | Disperse Yellow 241 |
| 340.14042 | C_17_H_17_N_5_O_3_ | Disperse Orange 31 |
| 345.15573 | C_17_H_20_N_4_O_4_ | Disperse Red 17 |
| 349.10619 | C_16_H_17_ClN_4_O_3_ | Disperse Red 13 |
| 349.14075 | C_18_H_16_N_6_O_2_ | Disperse Red 73 |
| 352.1768 | C_19_H_21_N_5_O_2_ | Disperse Orange 33 |
| 352.23834 | C_22_H_29_N_3_O | 2-(2H-benzotriazol-2-yl)-4,6-di-tert-pentylphenol |
| 353.13969 | C_22_H_16_N_4_O | Disperse Orange 13 |
| 357.13797 | C_18_H_20_N_4_O_2_S | Disperse Red 379 |
| 358.10653 | C_17_H_16_ClN_5_O_2_ | Disperse Red 50 |
| 358.16807 | C_20_H_24_ClN_3_O | 2,4-di-tert-butyl-6-(5-chlorobenzotriazol-2-yl)phenol |
| 361.15065 | C_17_H_20_N_4_O_5_ | Disperse Red 2 |
| 362.06506 | C_15_H_12_ClN_5_O_4_ | Disperse Yellow 211 |
| 363.09755 | C_20_H_14_N_2_O_5_ | Disperse Blue 35 (2) |
| 363.1564 | C_19_H_18_N_6_O_2_ | Disperse Blue 366 |
| 366.12305 | C_15_H_19_N_5_O_4_S | Disperse Blue 102 |
| 368.11018 | C_16_H_13_N_7_O_4_ | Disperse Orange 56 |
| 368.1387 | C_15_H_21_N_5_O_4_S | Disperse Blue 102 |
| 369.05157 | C_15_H_14_Cl_2_N_4_O_3_ | Disperse Orange 5 |
| 370.08877 | C_18_H_16_ClN_5_S | Disperse Red 153 |
| 370.15098 | C_18_H_19_N_5_O_4_ | Disperse Violet 33 |
| 372.12218 | C_18_H_18_ClN_5_O_2_ | Disperse Red 65 |
| 374.10144 | C_17_H_16_ClN_5_O_3_ | Disperse Red 54:1 |
| 376.14379 | C_17_H_21_N_5_O_3_S | Disperse Blue 124 |
| 378.11968 | C_19_H_15_N_5_O_4_ | Disperse Orange 29 |
| 378.12305 | C_16_H_19_N_5_O_4_S | Disperse Blue 124 |
| 379.11676 | C_17_H_19_ClN_4_O_4_ | Disperse Red 5 |
| 381.16697 | C_19_H_20_N_6_O_3_ | Disperse Violet 63 |
| 381.17099 | C_24_H_20_N_4_O | Sudan IV |
| 382.15098 | C_19_H_19_N_5_O_4_ | Disperse Orange 31 |
| 383.10178 | C_18_H_15_ClN_6_O_2_ | Disperse Orange 44 |
| 384.14887 | C_19_H_21_N_5_O_2_S | Disperse Red 107 |
| 392.06756 | C_17_H_15_Cl_2_N_5_O_2_ | Disperse Orange 37 |
| 393.16697 | C_20_H_20_N_6_O_3_ | Disperse Orange 80 |
| 395.12847 | C_19_H_18_N_6_O_2_S | Disperse Red 179 |
| 397.11743 | C_19_H_17_ClN_6_O_2_ | Disperse Orange 45 (component) ^†^ |
| 397.99728 | C_15_H_10_Cl_3_N_5_O_2_ | Disperse Orange 41 |
| 399.06214 | C_16_H_16_Cl_2_N_4_O_4_ | Disperse Brown 22 |
| 402.1772 | C_19_H_23_N_5_O_5_ | Disperse Violet 13 |
| 404.0498 | C_18_H_15_Cl_2_N_5_S | Disperse Red 153 |
| 406.16221 | C_20_H_19_N_7_O_3_ | Disperse Blue 165 |
| 407.11321 | C_16_H_18_N_6_O_5_S | Disperse Green 9 |
| 407.14623 | C_20_H_18_N_6_O_4_ | Disperse Red 72 |
| 409.0965 | C_20_H_16_N_4_O_4_S | Disperse Yellow 114 |
| 409.18388 | C_18_H_28_N_6_OS_2_ | Disperse Red 338 |
| 411.15977 | C_20_H_22_N_6_O_2_S | Disperse Red 343 |
| 414.12305 | C_19_H_19_N_5_O_4_S | Disperse Blue 148 |
| 416.07562 | C_18_H_14_ClN_5_O_5_ | Disperse Blue 85 |
| 416.11201 | C_19_H_18_ClN_5_O_4_ | Disperse Red 54, Disperse Red 54:1 |
| 417.06281 | C_18_H_14_Cl_2_N_6_O_2_ | Disperse Yellow 163 |
| 418.06545 | C_19_H_17_Cl_2_N_5_S | Disperse Red 152 |
| 424.23432 | C_23_H_29_N_5_O_3_ | Disperse Yellow 198 |
| 425.17205 | C_24_H_20_N_6_O_2_ | Disperse Red 184 |
| 426.07304 | C_17_H_17_Cl_2_N_5_O_4_ | Disperse Red 324 |
| 426.15204 | C_19_H_19_N_7_O_5_ | Disperse Blue 165:1 |
| 429.01856 | C_16_H_14_Cl_2_N_4_O_4_S | Disperse Yellow 108 |
| 429.14364 | C_20_H_21_ClN_6_O_3_ | Disperse Violet 63 |
| 429.17686 | C_21_H_24_N_4_O_6_ | Disperse Yellow 126 |
| 430.10021 | C_19_H_19_N_5_O_3_S_2_ | Disperse Red 137 |
| 433.02316 | C_16_H_15_Cl_3_N_4_O_4_ | Disperse Brown 1 |
| 433.13856 | C_19_H_21_ClN_6_O_4_ | Disperse Violet 93, Disperse Violet 93:1 |
| 434.19351 | C_22_H_23_N_7_O_3_ | Disperse Blue 165 |
| 439.1183 | C_20_H_18_N_6_O_4_S | Disperse Red 177 |
| 439.19107 | C_22_H_26_N_6_O_2_S | Disperse Red 343 (Dipropylamino) |
| 440.15646 | C_21_H_21_N_5_O_6_ | Disperse Red 82 |
| 441.18809 | C_21_H_24_N_6_O_5_ | Disperse Violet 77 |
| 444.16663 | C_24_H_21_N_5_O_4_ | Disperse Orange 73 |
| 445.18703 | C_25_H_24_N_4_O_4_ | Disperse Yellow 231 |
| 447.14451 | C_19_H_22_N_6_O_5_S | Disperse Blue 149 |
| 448.23834 | C_30_H_29_N_3_O | 2-(2H-benzotriazol-2-yl)-4,6-bis(1-methyl-1-phenylethyl)phenol |
| 450.07304 | C_19_H_17_Cl_2_N_5_O_4_ | Disperse Orange 30, Disperse Orange 61 |
| 454.17211 | C_22_H_23_N_5_O_6_ | Disperse Violet 33 |
| 459.07748 | C_19_H_19_BrN_6_O_3_ | Disperse Blue 183:1 |
| 459.17753 | C_24_H_22_N_6_O_4_ | Disperse Orange 49 |
| 465.12839 | C_19_H_21_ClN_6_O_6_ | Disperse Blue 291 (Cl) |
| 472.11077 | C_21_H_21_N_5_O_4_S_2_ | Disperse Red 136 |
| 472.18267 | C_22_H_25_N_5_O_7_ | Disperse Red 74 |
| 473.09313 | C_20_H_21_BrN_6_O_3_ | Disperse Blue 183 |
| 473.13347 | C_21_H_21_ClN_6_O_5_ | Disperse Red 202 |
| 474.11749 | C_21_H_20_ClN_5_O_6_ | Disperse Red 167 |
| 476.97265 | C_16_H_15_BrCl_2_N_4_O_4_ | Disperse Brown 4 |
| 479.06731 | C_18_H_19_BrN_6_O_5_ | Disperse Violet 93:1 |
| 479.96653 | C_17_H_15_Br_2_N_5_O_2_ | Disperse Orange 61 |
| 486.19832 | C_23_H_27_N_5_O_7_ | Disperse Red 74 |
| 488.05641 | C_20_H_18_BrN_5_O_5_ | Disperse Blue 321 |
| 489.12839 | C_21_H_21_ClN_6_O_6_ | Disperse Blue 291:1 (Cl) |
| 491.06731 | C_19_H_19_BrN_6_O_5_ | Disperse Blue 128 |
| 494.21464 | C_24_H_27_N_7_O_5_ | Disperse Blue 337 |
| 506.1437 | C_22_H_24_ClN_5_O_7_ | Disperse Red 167:1 |
| 509.07787 | C_19_H_21_BrN_6_O_6_ | Disperse Blue 291 |
| 517.16775 | C_22_H_24_N_6_O_9_ | Disperse Red 311 |
| 520.15935 | C_23_H_26_ClN_5_O_7_ | Disperse Red 167 |
| 527.03275 | C_20_H_20_BrClN_4_O_6_ | Disperse Brown 19 |
| 533.07787 | C_21_H_21_BrN_6_O_6_ | Disperse Blue 291:1, Disperse Blue 373 |
| 534.07312 | C_20_H_20_BrN_7_O_6_ | Disperse Blue 281 |
| 534.19832 | C_27_H_27_N_5_O_7_ | Disperse Red 135 |
| 545.22554 | C_27_H_28_N_8_O_5_ | Disperse Red 279 |
| 551.18849 | C_26_H_26_N_6_O_8_ | Disperse Blue 130 (component)^†^ |
| 570.98224 | C_20_H_20_Br_2_N_4_O_6_ | Disperse Brown 19 |
| 581.13935 | C_23_H_25_ClN_6_O_10_ | Disperse Blue 79:1 (Cl) |
| 585.14952 | C_26_H_25_ClN_6_O_8_ | Disperse Blue 130 (component)^†^ |
| 595.155 | C_24_H_27_ClN_6_O_10_ | Disperse Blue 79:1 |
| 625.08883 | C_23_H_25_BrN_6_O_10_ | Disperse Blue 79:1 |
| 639.10448 | C_24_H_27_BrN_6_O_10_ | Disperse Blue 79 |
| 693.10681 | C_30_H_24_N_6_O_10_S_2_ | Disperse Blue 29 |

### **SI-6 – Retention time prediction model for disperse dyes**

The retention time prediction model used as one of the data filtering steps in the suspect screening of dye compounds. Experimental retention times of standards run on the HPLC system is plotted against log P for 21 standard dyes. Retention times, log P and number of azo bonds are listed in the table below.

| **Dye** | **Molecular formula** | **# of azo bonds** | **RT**  **[min]** | **Log *P*** |
| --- | --- | --- | --- | --- |
| Disperse Black 2 | C_14_H_16_N_4_O | 1 | 7.95 | 2.7 |
| Disperse Red 17 | C_17_H_20_N_4_O_4_ | 1 | 9.78 | 2.6 |
| Disperse Blue 106 | C_14_H_17_N_5_O_3_S | 1 | 10.1 | 3.4 |
| Disperse Orange 3 | C_12_H_10_N_4_O_2_ | 1 | 10.2 | 3.2 |
| Disperse Brown 22 | C_16_H_16_Cl_2_N_4_O_4_ | 1 | 10.3 | 3.5 |
| Disperse Yellow 3 | C_15_H_15_N_3_O_2_ | 1 | 10.5 | 2.9 |
| Disperse Blue 35 | C_15_H_12_N_2_O_4_ | 0 | 11.1 | 3.2 |
| Disperse Red 5 | C_17_H_19_ClN_4_O_4_ | 1 | 11.1 | 3.3 |
| Disperse Red 1 | C_16_H_18_N_4_O_3_ | 1 | 11.4 | 3.3 |
| Disperse Blue 124 | C_16_H_19_N_5_O_4_S | 1 | 12.4 | 4.0 |
| Disperse Red 13 | C_16_H_17_ClN_4_O_3_ | 1 | 12.8 | 4.0 |
| Disperse Orange 25 | C_17_H_17_N_5_O_2_ | 1 | 12.9 | 3.7 |
| Disperse Blue 14 | C_16_H_14_N_2_O_2_ | 1 | 12.9 | 3.9 |
| Disperse Blue 337 | C_24_H_27_N_7_O_5_ | 1 | 13.3 | 2.7 |
| Sudan I-d_5_ (IS) | C_16_D_5_H_7_N_2_O | 1 | 14.1 | 4.1 |
| Disperse Orange 37 | C_17_H_15_Cl_2_N_5_O_2_ | 1 | 14.2 | 5.0 |
| Sudan I | C_16_H_12_N_2_O | 1 | 14.2 | 4.1 |
| Disperse Blue 79 | C_24_H_27_BrN_6_O_10_ | 1 | 14.3 | 3.5 |
| Disperse Orange 1 | C_18_H_14_N_4_O_2_ | 1 | 14.8 | 5.5 |
| Disperse Orange 13 | C_22_H_16_N_4_O | 2 | 16.7 | 6.4 |
| Sudan IV | C_24_H_20_N_4_O | 2 | 20.2 | 7.1 |

### **SI-7 – Instrumental HPLC/TSQ MS-MRM settings for disperse dyes**

Selected settings for the MRM-MS analysis are listed below: polarity mode, cone voltage, quantifier and qualifier ions, collision energy (CE), and dwell time.

| **Compound** | **Mw**  **[Da]** | **Polarity mode** | **Cone voltage**  **[V]** | **Quantifier ion** | | **Qualifier ion** | | **Dwell time**  **[ms]** |
| --- | --- | --- | --- | --- | --- | --- | --- | --- |
|  |  |  |  | **m/z** | **CE [eV]** | **m/z** | **CE [eV]** |  |
| Disperse Orange 3 | 242.1 | Positive | 2 | 75.3 | 28 | 122 | 18 | 20 |
| Disperse Yellow 3 | 269.1 | Positive | 2 | 150 | 16 | 122 | 14 | 20 |
| Disperse Orange 1 | 318.1 | Positive | 8 | 122 | 20 | 169.1 | 22 | 125 |
| Disperse Orange 37 | 391.1 | Positive | 2 | 133.1 | 34 | 351.1 | 20 | 20 |
| Disperse Orange 13 | 352.1 | Positive | 6 | 93 | 26 | 260.3 | 16 | 125 |
| Disperse Orange 25 | 323.1 | Positive | 2 | 122 | 36 | 283.1 | 18 | 126 |
| Disperse Blue 14 | 266.1 | Positive | 52 | 252 | 18 | 235 | 28 | 94 |
| Disperse Red 1 | 314.1 | Positive | 2 | 134.1 | 24 | 255.1 | 26 | 94 |
| Disperse Red 13 | 348.1 | Positive | 78 | 134.1 | 24 | 289 | 28 | 126 |
| Disperse Blue 124 | 377.1 | Positive | 2 | 87 | 26 | 220.1 | 16 | 126 |
| Disperse Blue 35 | 362.1 | Positive | 42 | 270.1 | 55 | 141.9 | 24 | 94 |
| Disperse Blue 106 | 335.1 | Positive | 5 | 147.2 | 30 | 178.1 | 16 | 20 |
| Disperse Red 17 | 344.1 | Positive | 2 | 164.1 | 26 | 269.1 | 28 | 20 |
| Sudan IV | 380.2 | Positive | 2 | 91 | 28 | 224.5 | 20 | 125 |
| Sudan I | 248.1 | Positive | 2 | 93 | 24 | 156 | 14 | 20 |
| Disperse Red 19 | 493.2 | Positive | 18 | 150.1 | 24 | 255.1 | 28 | 20 |
| Disperse Black 2 | 638.1 | Positive | 52 | 92 | 20 | 120 | 14 | 20 |
| Disperse Blue 337 | 398.1 | Positive | 10 | 191.1 | 24 | 120.9 | 64 | 126 |
| Disperse Blue 79 | 378.1 | Positive | 2 | 87 | 25 | 233.1 | 30 | 20 |
| Disperse Brown 22 | 253.1 | Positive | 3 | 118.9 | 40 | 163.1 | 28 | 20 |
| Disperse Red 5 | 378.8 | Positive | 2 | 177.1 | 28 | 164.2 | 26 | 20 |
| Methyl Orange | 327.3 | Negative | 22 | 156 | 26 | 225 | 26 | 250 |
| Sudan I-d_5_ (IS) | 253.3 | Positive | 4 | 98.8 | 20 | 156.1 | 18 | 20 |
| Disperse Red 60 | 331.3 | Positive | 24 | 127.1 | 58 | 238.9 | 30 | 20 |

### **SI-8 - Instrumental settings and calibration data for GC/MS analysis**

Quantifier and qualifier ions in full scan, slope, intercept and coefficient of determination (R^2^) for the linear calibration curves, as well as the RMSE and limit of detection (LOD), the latter given as the injected amount.

| **Compound** | **Quantifier ion** | **Qualifier ions** | **Slope** | **Intercept** | **R²** | **RMSE** | **LOD [pg]** |
| --- | --- | --- | --- | --- | --- | --- | --- |
| 4-chloroaniline | 127 | 92/65 | 0.21 | -0.08 | 0.9948 | 5.50% | 10.5 |
| 1,4-phenylenediamine | 108 | 80/53 | 0.1 | -0.02 | 0.8413 | 16.10% | 4.8 |
| 2-nitroaniline | 138 | 92/65 | 0.1 | -0.06 | 0.9923 | 3.20% | 25.3 |
| 3-nitroaniline | 92 | 92/138 | 0.34 | 0.03 | 0.9957 | 8.30% | 26.2 |
| 1-chloro-3,4-dinitrobenzene | 75 | 202/110 | 0.27 | -0.1 | 0.9953 | 6.80% | 23.5 |
| 1-chloro-2,4-dinitrobenzene | 75 | 202/110 | 0.19 | -0.1 | 0.9958 | 7.20% | 65.2 |
| 2,6-dichlorobenzene-1,4-diamine | 176 | 78/52 | 0.44 | -0.08 | 0.9951 | 11.30% | 8.9 |
| 5-chloro-2-nitroaniline | 172 | 142/99 | 0.23 | -0.04 | 0.9956 | 5.70% | 13.7 |
| 4-chloro-2-nitroaniline | 172 | 126/99 | 0.28 | -0.01 | 0.9953 | 7.00% | 10.4 |
| 4-nitroaniline | 138 | 65/108 | 0.28 | -0.09 | 0.9959 | 6.60% | 279.4 |
| 1-bromo-3,5-dinitrobenzene | 75 | 246/155 | 0.41 | -0.14 | 0.9957 | 10.20% | 24.3 |
| 2-chloro-4-nitroaniline | 172 | 90/142 | 0.22 | -0.1 | 0.9948 | 5.80% | 21.1 |
| 2,6-dichloro-4-nitroaniline | 124 | 206/176 | 0.2 | -0.03 | 0.9955 | 5.00% | 1.3 |
| 6-chloro-2,4-dinitroaniline | 217 | 125/90 | 0.14 | -0.15 | 0.9737 | 8.50% | 10 |
| 2,4-dinitroaniline | 183 | 153/107 | 0.14 | -0.2 | 0.9575 | 11.30% | 237 |
| 2-bromo-4,6-dinitroaniline | 261 | 231/169 | 0.04 | -0.04 | 0.9774 | 2.20% | 240.4 |

### **SI-9 – Disperse dyes detected in textile garments**

Detection frequencies of disperse dyes in clothing and theoretical precursors/cleavage products from reduction. Confirmation by reference compounds are marked with †. The “MS^2^” refers to when [M+H]^+^ has been extracted with 5 ppm mass accuracy, the LC retention time is within ±3 min from predicted, the isotopic pattern is within ±5 % from the theoretical relative abundance, and MS^2^ data are acquired. “No MS^2^” refers to the number of samples for which the ion intensities were too low to yield MS^2^-data. The frequency is based on the total detected, i.e. the sum of “MS^2^” and “No MS^2^”. Azo = the dye contains one azo-bond, 2 x azo = the dye contains 2 azo-bonds, anthraq. = anthraquinone-based dye.

| **Dye** | **CAS #** | **Dye type** | **Precursor/cleavage product** | **CAS #** |  | **MS^2^ (#)** | **No MS^2^ (#)** | **Frequency [%]** |
| --- | --- | --- | --- | --- | --- | --- | --- | --- |
| Disperse Black 1 | 6054-48-4 | azo | 1,4-phenylenediamine | 106-50-3 |  | 3 | 5 | 10 |
|  |  |  | naphthalene-1,4-diamine | 2243-61-0 |  |  |  |  |
| ^†^Disperse Black 2 | 6232-57-1 | azo | 1,4-phenylenediamine | 106-50-3 |  | 2 | 0 | 2 |
|  |  |  | 2-methoxy-5-methylbenzene-1,4-diamine | 5307-00-6 |  |  |  |  |
| ^†^Disperse Blue 14 | 2475-44-7 | anthraq. | - | - |  | 6 | 0 | 7 |
| ^†^Disperse Blue 35 | 12222-75-2 | anthraq. | - | - |  | 6 | 0 | 7 |
| ^†^Disperse Blue 79  (Br) | 12239-34-8 | azo | 2-bromo-4,6-dinitroaniline | 1817-73-8 |  | 4 | 0 | 5 |
|  |  |  | ((5-acetamido-4-amino-2-ethoxyphenyl) azanediyl)bis(ethane-2,1-diyl) diacetate | - |  |  |  |  |
| Disperse Blue 79  (Cl) | 3618-73-3 | azo | 2-chloro-4,6-dinitroaniline | 3531-19-9 |  | 7 | 6 | 16 |
|  |  |  | ((5-acetamido-4-amino-2-methoxyphenyl) azanediyl)bis(ethane-2,1-diyl) diacetate | - |  |  |  |  |
| Disperse Blue 149 | 32569-24-7 | azo | 5-nitro-3H-2l4-benzo[c]isothiazol-3-amine | - |  | 1 | 0 | 1 |
|  |  |  | N-(2-amino-5-(bis(2-hydroxyethyl)amino) phenyl)acetamide | - |  |  |  |  |
| Disperse Blue 165 | 56532-53-7 | azo | 2-amino-5-nitroisophthalonitrile | - |  | 6 | 16 | **27** |
|  |  |  | N-(2-amino-5-(dipropylamino)phenyl) acetamide | - |  |  |  |  |
| Disperse Blue 165:1 | 24170-60-3 | azo | 2-amino-3,5-dinitrobenzonitrile | 22603-53-8 |  | 2 | 2 | 5 |
|  |  |  | N-(2-amino-5-(diethylamino)phenyl) acetamide | 2359-47-9 |  |  |  |  |
| Disperse Blue 183 | 2309-94-6 | azo | 2-amino-3-bromo-5-nitrobenzonitrile | 17601-94-4 |  | 1 | 13 | 17 |
|  |  |  | N-(2-amino-5-(diethylamino)phenyl) propionamide | - |  |  |  |  |
| Disperse Blue 183:1 | 2537-62-4 | azo | 2-amino-3-bromo-5-nitrobenzonitrile | 17601-94-4 |  | 12 | 6 | **22** |
|  |  |  | N-(2-amino-5-(diethylamino)phenyl) acetamide | 2359-47-9 |  |  |  |  |
| Disperse Blue 291 (Br) | 56548-64-2 | azo | 2-bromo-4,6-dinitroaniline | 1817-73-8 |  | 2 | 7 | 11 |
|  |  |  | N-(2-amino-5-(diethylamino)-4-methoxy phenyl)acetamide | - |  |  |  |  |
| Disperse Blue 291 (Cl) | 79295-99-1 | azo | 6-chloro-2,4-dinitroaniline | 3531-19-9 |  | 11 | 10 | **26** |
|  |  |  | N-(2-amino-5-(diethylamino)-4-methoxy phenyl)acetamide | - |  |  |  |  |
| ^†^Disperse Blue 337 | 65916-12-3 | azo | 2-amino-5-nitroisophthalonitrile | 20033-48-1 |  | 1 | 0 | 1 |
|  |  |  | N-(2-amino-5-((2-(2-ethoxyethoxy)ethyl) (ethyl) amino)phenyl)acetamide | - |  |  |  |  |
| Disperse Blue 366 | 84870-65-5 | azo | 4-diethylamino-o-toluidine | 148-71-0 |  | 10 | 8 | **22** |
|  |  |  | 2-amino-5-nitroisophthalonitrile | 20033-48-1 |  |  |  |  |
| Disperse Brown 19 | 55619-18-6 | azo | 2,6-dibromo-4-nitroaniline | 827-94-1 |  | 1 | 16 | **21** |
|  |  |  | ((4-aminophenyl)azanediyl)bis(ethane-2,1-diyl) diacetate | - |  |  |  |  |
| ^†^Disperse Brown 22 | 63467-07-2 | azo | 2,6-dichloro-4-nitroaniline | 99-30-9 |  | 6 | 0 | 7 |
|  |  |  | 2,2'-((4-aminophenyl)azanediyl)bis(ethan-1-ol) | - |  |  |  |  |
| Disperse Green 9 | 58979-46-7 | azo | 2-amino-3,5-dinitrothiophene | 2045-70-7 |  | 6 | 1 | 9 |
|  |  |  | N-(2-amino-5-(diethylamino)phenyl) acetamide | 2359-47-9 |  |  |  |  |
| Disperse Orange 1.1 | 26311-09-1 | azo | 4-nitroaniline | 100-01-6 |  | 1 | 2 | 4 |
|  |  |  | N-(2,5-diaminophenyl)acetamide | - |  |  |  |  |
| ^†^Disperse Orange 3 | 730-40-5 | azo | 1,4-phenylenediamine | 106-50-3 |  | 21 | 0 | **26** |
|  |  |  | 4-nitroaniline | 100-01-6 |  |  |  |  |
| ^†^Disperse Orange 25 | 31482-56-1 | azo | 4-nitroaniline | 100-01-6 |  | 32 | 0 | **39** |
|  |  |  | 3-((4-aminophenyl)(ethyl)amino) propanenitrile | - |  |  |  |  |
| Disperse Orange 29 | 19800-42-1 | 2 x azo | 4-amino-phenol | 123-30-8 |  | 2 | 1 | 4 |
|  |  |  | 1,4-diamino-2-methoxybenzene | 5307-02-08 |  |  |  |  |
|  |  |  | 4-nitroaniline | 100-01-6 |  |  |  |  |
| Disperse Orange 33 | 69472-19-1 | azo | 4-nitroaniline | 100-01-6 |  | 3 | 2 | 6 |
|  |  |  | 3-((4-(4-aminophenyl)butyl)amino) propanenitrile | - |  |  |  |  |
| ^†^Disperse Orange 37 | 13301-61-6 | azo | 2,6-dichloro-4-nitroaniline | 99-30-9 |  | 8 | 0 | 10 |
|  |  |  | 2,2'-((4-aminophenyl)azanediyl)  bis(ethan-1-ol) | - |  |  |  |  |
| Disperse Orange 44 | 4058-30-4 | azo | 2-chloro-4-nitroaniline | 121-87-9 |  | 4 | 5 | 11 |
|  |  |  | 3,3'-((4-aminophenyl)azanediyl) dipropanenitrile | - |  |  |  |  |
| Disperse Orange 61 | 55281-26-0 | azo | 2,6-dibromo-4-nitroaniline | 827-94-1 |  | 2 | 14 | 20 |
|  |  |  | 3-((4-aminophenyl)(ethyl)amino) propanenitrile | - |  |  |  |  |
| Disperse Orange 73 | 40690-89-9 | azo | 4-nitroaniline | 100-01-6 |  | 5 | 3 | 10 |
|  |  |  | 2-((4-aminophenyl)(2-cyanoethyl)amino) ethyl benzoate | - |  |  |  |  |
| Disperse Orange 80 | 70210-10-5 | azo | 4-nitroaniline | 100-01-6 |  | 1 | 5 | 7 |
|  |  |  | 3-((4-aminophenyl)(2-(2-cyanoethoxy)ethyl)amino)propanenitrile | - |  |  |  |  |
| ^†^Disperse Red 1 | 2872-52-8 | azo | 4-nitroaniline | 100-01-6 |  | 5 | 0 | 6 |
|  |  |  | 2-((4-aminophenyl)(ethyl)amino)  ethan-1-ol | 1225351-20-1 |  |  |  |  |
| Disperse Red 2 | 3769-58-2 | azo | 4-nitroaniline | 100-01-6 |  | 1 | 0 | 1 |
|  |  |  | 3-((4-aminophenyl)(2hydroxyethyl)amino) propane-1,2-diol | - |  |  |  |  |
| ^†^Disperse Red 13 | 3180-81-2 | azo | 2-chloro-4-nitroaniline | 121-87-9 |  | 4 | 0 | 5 |
|  |  |  | 2-((4-aminophenyl)(ethyl)amino)  ethan-1-ol | 1225351-20-1 |  |  |  |  |
| ^†^Disperse Red 17 | 3179-89-3 | azo | 4-nitroaniline | 100-01-6 |  | 3 | 0 | 4 |
|  |  |  | 2,2'-((4-amino-3-methylphenyl)azanediyl) bis(ethan-1-ol) | - |  |  |  |  |
| ^†^Disperse Red 19 | 2734-52-3 | azo | 4-nitroaniline | 100-01-6 |  | 7 | 0 | 9 |
|  |  |  | 2,2'-((4-aminophenyl)azanediyl)  bis(ethan-1-ol) | - |  |  |  |  |
| Disperse Red 50 | 40880-51-1 | azo | 2-chloro-4-nitroaniline | 121-87-9 |  | 4 | 2 | 7 |
|  |  |  | 3-((4-aminophenyl)(ethyl)amino) propanenitrile | - |  |  |  |  |
| Disperse Red 54:1 | 6657-33-6 | azo | 2-chloro-4-nitroaniline | 121-87-9 |  | 2 | 8 | 12 |
|  |  |  | 3-((4-aminophenyl)(2-hydroxyethyl) amino)propanenitrile | - |  |  |  |  |
| ^†^Disperse Red 60 | 17418-58-5 | anthraq. | - |  |  | 31 |  | **38** |
| Disperse Red 65 | 16586-43-9 | azo | 2-chloro-4-nitroaniline | 121-87-9 |  | 5 | 2 | 9 |
|  |  |  | 3-((4-amino-3-methylphenyl)(ethyl)amino propanenitrile | - |  |  |  |  |
| Disperse Red 72 | 12223-39-1 | azo | 2-amino-5-nitrobenzonitrile | 17420-30-3 |  | 1 | 0 | 1 |
|  |  |  | 2-((4-aminophenyl)(2-cyanoethyl)amino)ethyl acetate | - |  |  |  |  |
| Disperse Red 73 | 16889-10-4 | azo | 2-amino-5-nitrobenzonitrile | 17420-30-3 |  | 7 | 12 | **23** |
|  |  |  | 3-((4-aminophenyl)(ethyl)amino) propanenitrile | - |  |  |  |  |
| Disperse Red 74 | 1533-74-0 | azo | 4-nitroaniline | 100-01-6 |  | 20 | 16 | **44** |
|  |  |  | ((3-acetamido-4-aminophenyl)azanediyl) bis(ethane-2,1-diyl) diacetate | - |  |  |  |  |
| Disperse Red 82 | 30124-94-8 | azo | 2-amino-5-nitrobenzonitrile | 17420-30-3 |  | 4 | 1 | 6 |
|  |  |  | ((4-aminophenyl)azanediyl)bis(ethane-2,1-diyl) diacetate | - |  |  |  |  |
| Disperse Red 152 | 78520-83-9 | azo | 2-amino-5,6-dichlorobenzothiazole | 25150-27-0 |  | 6 | 0 | 7 |
|  |  |  | 3-((4-amino-3-methylphenyl) (ethyl)amino)propanenitrile | - |  |  |  |  |
| Disperse Red 153 | 25150-28-1 | azo | 5,7-dichlorobenzo[d]thiazol-2-amine | - |  | 21 | 17 | **46** |
|  |  |  | 3-((4-aminophenyl)(ethyl)amino) propanenitrile | - |  |  |  |  |
| Disperse Red 167 | 26850-12-4 | azo | 2-chloro-4-nitroaniline | 121-87-9 |  | 7 | 4 | 13 |
|  |  |  | ((4-amino-3-propionamidophenyl) azanediyl)bis(ethane-2,1-diyl) diacetate | - |  |  |  |  |
| Disperse Red 167:1 | 79300-13-3 | azo | 2-chloro-4-nitroaniline | 121-87-9 |  | 26 | 29 | **67** |
|  |  |  | ((3-acetamido-4-aminophenyl)azanediyl) bis(ethane-2,1-diyl) diacetate | - |  |  |  |  |
| Disperse Red 177 | 58051-98-2 | azo | 2-amino-6-nitrobenzothiazole | 6285-57-0 |  | 1 | 4 | 6 |
|  |  |  | 2-((4-aminophenyl)(2-cyanoethyl)amino)ethyl acetate | - |  |  |  |  |
| Disperse Red 179 | 16586-42-8 | azo | 2-amino-6-nitrobenzothiazole | 6285-57-0 |  | 5 | 8 | 16 |
|  |  |  | 3-((4-amino-3-methylphenyl)(ethyl) amino)propanenitrile | - |  |  |  |  |
| Disperse Red 202 | 28462-17-1 | azo | 2-chloro-4-nitroaniline | 121-87-9 |  | 8 | 4 | 15 |
|  |  |  | 2-((3-acetamido-4-aminophenyl)(2-cyano ethyl)amino)ethyl acetate | - |  |  |  |  |
| Disperse Red 311 | 70729-65-6 | azo | 2,4-dinitroaniline | 97-02-09 |  | 16 | 23 | **48** |
|  |  |  | dimethyl 3,3'-((3-acetamido-4-amino phenyl)azanediyl)dipropionate | - |  |  |  |  |
| Disperse Red 324 | 71617-28-2 | azo | 2-chloro-4-nitroaniline | 121-87-9 |  | 2 | 5 | 9 |
|  |  |  | N-(2-amino-4-chloro-5-((2-hydroxy propyl)amino)phenyl)acetamide | - |  |  |  |  |
| Disperse Red 343 | 68385-96-6 | azo | 2-amino-5-methylisophthalonitrile | - |  | 17 | 23 | **49** |
|  |  |  | N-(2-amino-5-(diethylamino)phenyl)methanesulfon amide | - |  |  |  |  |
| Disperse Violet 63 | 52583-54-7 | azo | 2-amino-5-nitrobenzonitrile | 17420-30-3 |  | 10 | 7 | **21** |
|  |  |  | N-(2-amino-6-(diethylamino)phenyl)acetamide | - |  |  |  |  |
| Disperse Violet 77 | 52549-57-2 | azo | 2-amino-5-nitrobenzonitrile | 17420-30-3 |  | 2 | 5 | 9 |
|  |  |  | N-(2-amino-5-(bis(2-methoxyethyl)amino) phenyl)acetamide | - |  |  |  |  |
| Disperse Violet 93:1 | 52697-38-8 | azo | 2-bromo-4,6-dinitroanilin | 1817-73-8 |  | 6 | 3 | 11 |
|  |  |  | N-(2-amino-5-(diethylamino)phenyl) acetamide | 2359-47-9 |  |  |  |  |
| ^†^Disperse Yellow 3 | 2832-40-8 | azo | 2-amino-4-methylphenol | 95-84-1 |  | 7 | 0 | 9 |
|  |  |  | N-(4-aminophenyl)acetamide | 122-80-5 |  |  |  |  |
| Disperse Yellow 119 | 49744-25-4 | azo | 2-nitroaniline | 88-74-4 |  | 4 | 0 | 5 |
|  |  |  | 5-amino-1-ethyl-6-hydroxy-4-methyl-2-oxo-1,2-dihydropyridine-3-carbonitrile | - |  |  |  |  |
| Disperse Yellow 126 | 49744-26-5 | azo | 2-(2-methoxyethoxy)ethyl-anthranilate | 49744-35-6 |  | 1 | 1 | 2 |
|  |  |  | 5-amino-1-ethyl-6-hydroxy-4-methyl-2-oxo-1,2-dihydropyridine-3-carbonitrile | - |  |  |  |  |
| Disperse Yellow 163 | 67923-43-7 | azo | 2,6-dichloro-4-nitroaniline | 99-30-9 |  | 2 | 0 | 2 |
|  |  |  | 3,3'-((4-aminophenyl)azanediyl)dipropane nitrile | - |  |  |  |  |
| Disperse Yellow 211 | 70528-90-4 | azo | 4-chloro-2-nitroaniline | 89-63-4 |  | 4 | 6 | 12 |
|  |  |  | 5-amino-1-ethyl-6-hydroxy-4-methyl-2-oxo-1,2-dihydropyridine-3-carbonitrile | - |  |  |  |  |
| Disperse Yellow 231 | 75199-13-2 | azo | benzyl p-aminobenzoate | 19008-43-6 |  | 1 | 0 | 1 |
|  |  |  | 5-amino-1-butyl-6-hydroxy-4-methyl-2-oxo-1,2-dihydropyridine-3-carbonitrile | - |  |  |  |  |
| Disperse Yellow 241 | 83249-52-9 | azo | 3,4-dichloroaniline | 95-76-1 |  | 1 | 1 | 2 |
|  |  |  | 5-amino-6-hydroxy-1,4-dimethyl-2-oxo-1,2-dihydropyridine-3-carbonitrile | - |  |  |  |  |

### **SI-10 – Disperse dyes quantified in textile garments**

Quantified disperse dyes for which reference compounds were available.

| **Compound** | **Frequency**  **[%]** | **Concentration [µg/g]** | | | |
| --- | --- | --- | --- | --- | --- |
|  |  | **Min** | **Max** | **Mean** | **Median** |
| **Azo type dyes** | | | | |  |
| Disperse Orange 25 | 39 | 0.0073 | 82 | 8.0 | 0.16 |
| Disperse Orange 3 | 26 | 0.0054 | 3.7 | 0.46 | 0.049 |
| Disperse Orange 37 | 10 | 0.0049 | 1.6 | 0.32 | 0.036 |
| Disperse Red 19 | 9 | 0.012 | 1.0 | 0.25 | 0.040 |
| Disperse Yellow 3 | 9 | 0.011 | 0.075 | 0.037 | 0.024 |
| Disperse Brown 22 | 7 | 0.015 | 1.3 | 0.37 | 0.095 |
| Disperse Red 1 | 6 | 0.00067 | 0.079 | 0.043 | 0.033 |
| Disperse Blue 79 | 5 | 0.016 | 11 | 4.9 | 4.3 |
| Disperse Red 13 | 5 | 0.0056 | 0.12 | 0.04 | 0.019 |
| Disperse Red 17 | 4 | 0.027 | 0.058 | 0.038 | 0.029 |
| Disperse Black 2 | 2 | 0.011 | 0.014 | 0.013 | 0.013 |
| **Anthraquinone type dyes** | | | | |  |
| Disperse Red 60 | 38 | 0.019 | 540 | 50 | 1.2 |
| Disperse Blue 14 | 7 | 0.0016 | 14 | 2.3 | 0.033 |
| Disperse Blue 35 | 7 | 0.025 | 23 | 8.6 | 4.6 |
